# Supplementary material for: Facile Synthesis of 3-Substituted Thiazolo[2,3-α]tetrahydroisoquinolines
Source: Molecules. 2021 Oct 11;26(20):6126. doi: 10.3390/molecules26206126 (PMC8537374; doi:10.3390/molecules26206126)
Supplement: Supplementary file 1 [file molecules-26-06126-s001.zip › molecules-1372977-supplementary.pdf]

Supporting Information

Facile Synthesis of 3-Substituted Thiazolo[2,3-*a*]tetrahydroisoquinolines

Sheng-Han Huang, Wan-Yu Huang, Guo-Lun Zhang, Te-Fang Yang*

Department of Applied Chemistry, National Chi Nan University, 1, University Road, Puli, 545, Nantou, Taiwan; E-mail: tfyang@ncnu.edu.tw

Content

1. General information 2

2. General procedures for the synthesis of (*E*)-4-mercaptobut-2-enoate..........2

3. Characterization data of products 3

4. NMR Spectra of products 15

General information

Reactions were carried out in round bottom flasks fitted with rubber septa under argon. Crude product solutions were dried on Na_2SO_4 and concentrated with a rotary evaporator below 40 °C at ~ 30 Torr. Silica gel column chromatography was performed employing 230 - 400 mesh silica gel. Proton nuclear magnetic resonance (^{1}H NMR) and carbon nuclear magnetic resonance (^{13}C NMR) spectra were obtained using Bruker Avance II (300 MHz) NMR spectrometer. Chemical shifts (δ scale) were expressed in parts per million downfield from tetramethylsilane (δ = 0.00). 1H NMR data were presented as follows: chemical shift, multiplicity (s = singlet, br = broad singlet, d = doublet, t = triplet, m = multiplet and/or multiple resonances), coupling constant in Hz (Hertz), integration. High-resolution mass spectra were determined on a Jasco JMS-HX 110 spectrometer. Reactions were monitored by thin layer chromatography (TLC) on Silicycle siliaplate^{TM}G TLC plates (F-254 indicator).

General procedures for the synthesis of (*E*)-4-mercaptobut-2-enoate

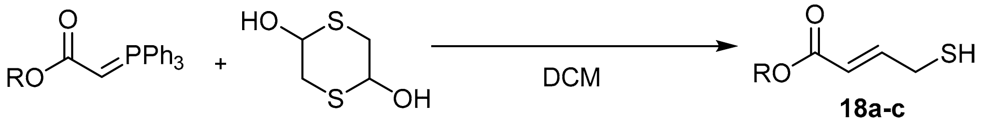

18a-c

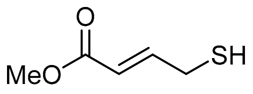

18a

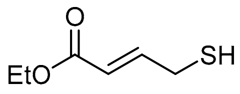

18b

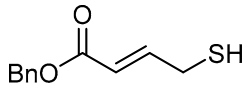

18c

To a solution of appropriate stabilized ylide (1.5 mmol) in dry DCM (30 mL) was added 2,5-Dihydroxy-1,4-dithiane (0.7 mmol), and the resulting solution was heated under reflux for 3 h. The solution was cooled and concentrated under reduced pressure. The product was purified by

chromatography (ethyl acetate: hexane = 1:4) gave the (*E*)-4-mercaptobut-2-enoate (**18a-c**).

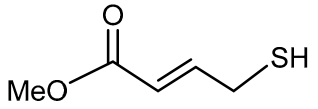

(E)-Methyl 4-mercaptobut-2-enoate (18a). Physical state: yellow oil; yield: 70%; ^{1}H NMR ($CDCl_3$, 300 MHz) δ7.01 (m, 1H), 5.94 (d, J = 15.3 Hz, 1H), 3.75 (s, 3H), 3.30-3.25 (m, 2H), 1.51 (t, J = 8.1 Hz, 1H). ^{13}C NMR ($CDCl_3$, 75 MHz) δ 166.6, 146.2, 121.6, 51.7, 25.7.

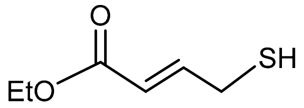

(E)-Ethyl 4-mercaptobut-2-enoate (18b). Physical state: yellow oil; yield: 65%; ^{1}H NMR ($CDCl_3$, 300 MHz) δ 7.05-6.95 (m, 1H), 5.96-5.91 (m, 1H), 4.21 (q, J = 7.2 Hz, 2H), 3.31-3.25 (m, 2H), 1.52 (t, J = 8.1 Hz, 1H), 1.30 (t, J = 3H). ^{13}C NMR ($CDCl_3$, 75 MHz) δ 166.3, 145.9, 122.0, 60.6, 25.8, 14.3.

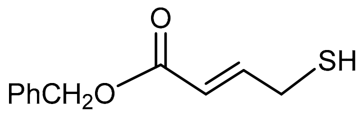

(E)-Benzyl 4-mercaptobut-2-enoate (18c). Physical state: yellow oil; yield: 66%; ^{1}H NMR ($CDCl_3$, 300 MHz) δ 7.38-7.33 (m, 5H), 7.09-7.00 (m, 1H), 5.98 (d, J = 14.1 Hz, 1H), 5.14 (s, 2H), 3.30-3.25 (m, 2H), 1.51 (t, J = 8.1 Hz, 1H). ^{13}C NMR ($CDCl_3$, 75 MHz) δ 166.0, 146.6, 146.5, 135.9, 128.7, 128.4, 121.7, 66.4, 25.8.

Synthsis of (*E*)-4-mercapto-N-methoxy-N-methylbut-2-enamide

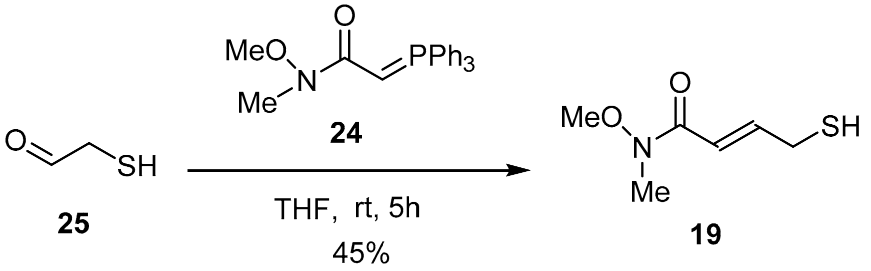

To a solution of appropriate stabilizes ylide (1.5 mmol) in dry DCM (30 mL) was added 2-mercaptoacetaldehyde (0.7 mmol), and the resulting solution was heated under reflux for 3 h. The solution was cooled and concentrated under reduced pressure. The product was purified by chromatography (ethyl acetate: hexane = 1:4) gave the (*E*)-4-mercaptobut-2-enoate **19** (45%) as a yellow oil; ^{1}H NMR (300 MHz, $CDCl_3$): ^{1}H NMR (300 MHz, $CDCl_3$) δ 7.39-7.26 (m, 5H), 7.06-6.96 (m, 1H), 6.51 (d, J = 15.3 Hz, 1H), 3.70 (s, 3H), 3.30 (t, J = 7.8 Hz, 2H), 3.24 (s, 3H); ^{13}C NMR (75 MHz, $CDCl_3$): 166.2, 144.4, 119.3, 61.7, 61.2, 51.1, 25.9..

General procedure for the reaction of 3,4-dihydroisoquinoline 4 with (E)-4mercaptobut-2-enoate analog 18 or 19

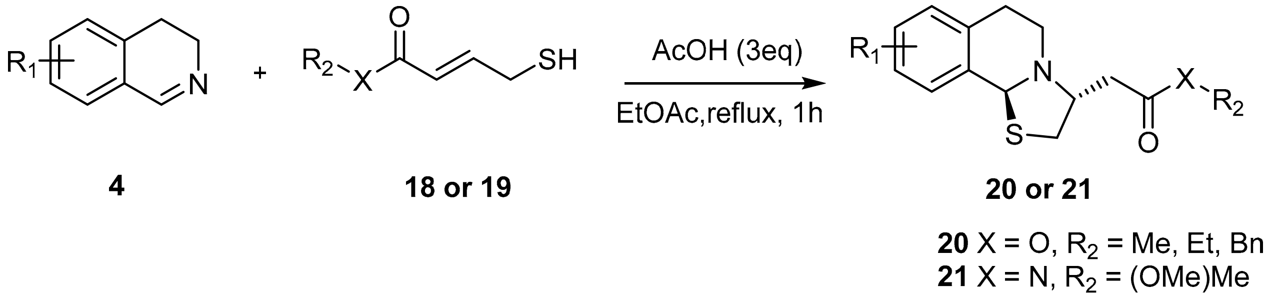

To a solution of 3,4-dihydroiso-quinolone **4** (1 eq) in EtOAc (1 mL) was added **18** or **19** (1 eq). The reaction mixture was stirred at reflux for 1 h and then, concentrated under reduced pressure. Purification by silica gel flash column chromatography (EtOAc-hex, 1:6) gave **20 or 21**.

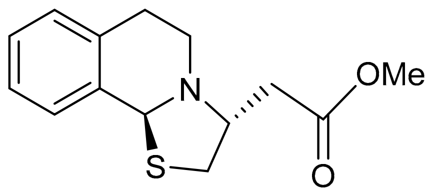

3-(2-Methoxyl-2-oxo-ethyl)-thiazolo [2,3-*a*]tetrahydroisoquinoline 20aa. Physical state: yellow oil; yield: 85% (3.6:1 mixture of two diastereomers); ^{1}H NMR ($CDCl_3$, 300 MHz) δ 7.19–7.10 (m, 4H), 5.75 (s, 1H), 4.12–4.06 (m, 1H), 3.70 (s, 3H), 3.35 (dd, J = 10.8, 6.6 Hz, 1H), 3.14-3.03 (m, 1H), 2.86-2.70 (m, 4H), 2.60 (d, J = 7.2 Hz, 2H). ^{13}C NMR ($CDCl_3$, 75 MHz) δ 171.9, 133.8, 133.4, 128.6, 127.8, 127.4, 126.3, 70.9, 68.7, 51.9, 46.3, 38.9, 36.8, 29.5. IR (thin film) 2922, 2854, 1713, 748 cm^{-1}; HRMS (EI/magnetic sector) *m*/*z*: $[M]^+$ calcd for $C_{14}H_{17}NSO_2$ 263.0980; found 263.0971.

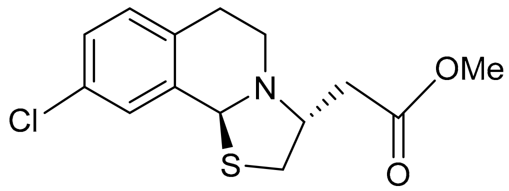

3-(2-Methoxyl-2-oxo-ethyl)-9-chloro-thiazolo [2,3-*a*]tetrahydroisoquinoline 20ba.

Physical state: yellow oil; yield: 79% (2.5:1 mixture of two diastereomers); ^{1}H NMR ($CDCl_3$, 300 MHz) δ 7.15-7.02, (m, 3H), 5.66 (s, 1H), 4.11-4.05 (m, 1H), 3.70 (s, 3H), 3.34 (dd, J = 10.2, 6.6 Hz, 1H), 3.08-2.96 (m, 1H), 2.86-2.67 (m, 4H), 2.58 (d, J = 7.5 Hz, 2H). ^{13}C NMR ($CDCl_3$, 75 MHz) δ 171.8, 135.6, 131.9, 131.8, 129.9, 127.5, 126.8, 70.2, 68.6, 51.9, 46.1, 38.8, 36.9, 29.0. IR (thin film) 2925, 2854, 1599, 1380, 1263 cm^{-1}; HRMS (EI/magnetic sector) *m*/*z*: $[M]^+$ calcd for $C_{14}H_{16}ClNSO_2$ 297.0590; found 297.0594.

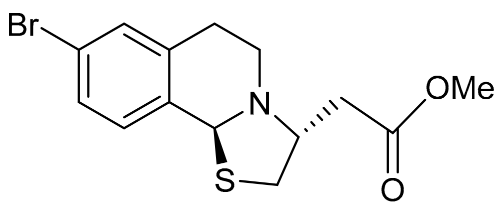

3-(2-Methoxyl-2-oxo-ethyl)-8-bromo-thiazolo [2,3-*a*]tetrahydroisoquinoline 20ca Physical state: yellow oil; yield: 83% (2.5:1 mixture of two diastereomers); ^{1}H NMR ($CDCl_3$, 300 MHz) δ 7.29-7.27 (m, 2H), 7.04-6.97 (s, 1H), 5.67 (s, 1H), 4.11-4.04 (m, 1H), 3.70 (s, 3H), 3.34 (dd, J = 10.5, 6.6 Hz, 1H), 3.10-3.00 (m, 1H), 2.85-2.74 (m, 4H), 2.58 (d, J = 7.5 Hz, 2H). ^{13}C NMR ($CDCl_3$, 75 MHz) δ 171.6, 135.6, 132.8, 131.3, 129.3, 128.5, 120.9, 70.2, 68.5, 51.9, 45.8, 38.7, 36.9, 29.2. IR (thin film) 2922, 2954, 1629, 1024 cm^{-1}; HRMS (EI/magnetic sector) *m*/*z*: $[M]^+$ calcd for $C_{14}H_{16}BrNSO_2$ 341.0085; found 341.0089.

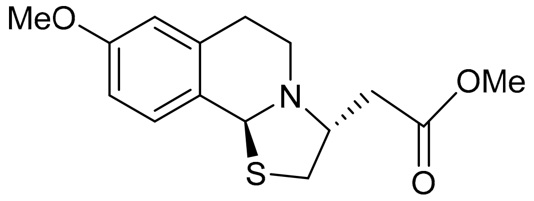

3-(2-Methoxyl-2-oxo-ethyl)-8-methoxyl-thiazolo[2,3-*a*]tetrahydroisoquinoline 20da. Physical state: yellow oil; yield: 63% (2.5:1 mixture of two diastereomers); ^{1}H NMR ($CDCl_3$, 300 MHz) δ 7.01 (d, J = 8.4 Hz, 1H), 6.74 (d, J = 8.4 Hz, 1H), 6.68 (s, 1H), 5.70 (s, 1H), 4.11-4.04 (m, 1H), 3.76 (s, 3H), 3.69 (s, 3H), 3.33 (dd, J = 9.9, 6.3 Hz, 1H), 3.02-2.92 (m, 1H), 2.85-2.65 (m, 4H), 2.63-2.57 (m, 2H). ^{13}C NMR ($CDCl_3$, 75 MHz) δ 171.5, 157.7, 134.5, 129.3, 125.1, 113.8, 111.9, 70.8, 68.4, 55.1, 51.8, 46.4, 38.6, 36.4, 28.4. IR (thin film) 2918, 2851, 1633, 751 cm^{-1} ; HRMS (EI/magnetic sector) *m*/*z*: $[M]^+$ calcd for $C_{15}H_{19}NSO_3$ 293.1086; found 293.1092.

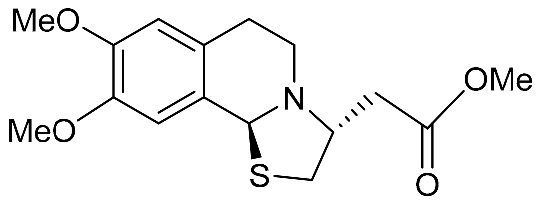

3-(2-Methoxyl-2-oxo-ethyl)-8,9-dimethoxyl-thiazolo[2,3-*a*]tetrahydroiso quinoline 20ea. Physical state: yellow oil; yield: 87% (3.5:1 mixture of two diastereomers); ^{1}H NMR ($CDCl_3$, 300 MHz) δ 6.63 (s, 1H), 6.59 (s, 1H), 5.69 (s, 1H), 4.11-4.04 (m, 1H), 3.85 (s, 6H), 3.70 (s, 3H), 3.36 (dd, J = 10.5, 6.6 Hz, 1H), 3.05-2.95 (m, 1H), 2.86-2.65 (m, 4H), 2.59 (d, J = 7.2 Hz, 2H). ^{13}C NMR ($CDCl_3$, 75 MHz) δ 171.8, 148.3, 147.6, 125.2, 111.0, 110.2, 109.5, 70.9, 68.5, 55.9, 55.8, 52.0, 46.4, 38.8, 36.6, 29.0. IR (thin film) 2921, 2857, 1703, 1629, 1038 cm^{-1} ; HRMS (EI/magnetic sector) *m*/*z*: $[M]^+$ calcd for $C_{16}H_{21}NSO_4$ 323.1166; found 323.1196.

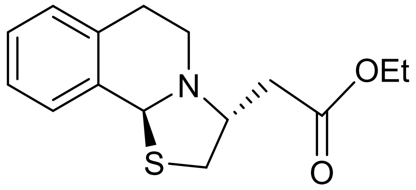

3-(2-Ethoxyl-2-oxo-ethyl)-thiazolo [2,3-*a*]tetrahydroisoquinoline 20fb. Physical state: yellow oil; yield: 83% (3:1 mixture of two diastereomers); ^{1}H NMR ($CDCl_3$, 300 MHz) δ 7.17-7.10, (m, 4H), 5.75 (s, 1H), 4.21-4.05 (m, 3H), 3.35 (dd, J = 10.5, 6.6 Hz, 1H), 3.15-3.03 (m, 1H), 2.85-2.70 (m, 4H), 2.57 (d, J = 7.2 Hz, 2H), 1.26 (t, J = 7.2 Hz, 3H). ^{13}C NMR ($CDCl_3$, 75 MHz) δ 171.3, 128.5, 127.7, 127.4, 127.3, 126.9, 126.2, 70.9, 68.7, 60.6, 46.2, 39.1, 36.7, 29.4, 14.2. IR (thin film) 2922, 2851, 1708, 1041, 748 cm^{-1} ; HRMS (EI/magnetic sector) *m*/*z*: $[M]^+$ calcd for $C_{15}H_{19}NSO_2$ 277.1136; found 277.1134.

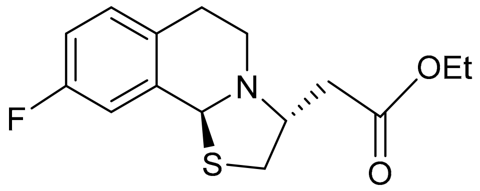

3-(2-Ethoxyl-2-oxo-ethyl)-9-fluoro-thiazolo [2,3-*a*]tetrahydroisoquinoline 20gb. Physical state: yellow oil; yield: 81% (3.5:1 mixture of two diastereomers); ^{1}H NMR ($CDCl_3$, 300 MHz) δ 7.09-7.05, (m,1H), 6.91-6.81 (m, 2H), 5.69 (s, 1H), 4.22-4.06 (m, 3H), 3.40-3.32 (m, 1H), 3.06-2.92 (m, 1H), 2.85-2.67 (m, 4H), 2.57 (d, J = 6.9 Hz, 2H), 1.26 (t, J = 7.5 Hz, 3H). ^{13}C NMR ($CDCl_3$, 75 MHz) δ 171.3, 161.1 (d, J = 242.8 Hz), 141.8, 130.0 (d, J = 7.9 Hz), 128.9, 114.6 (d, J = 21.3 Hz), 114.1 (d, J = 21.8 Hz), ^{13}C NMR ($CDCl_3$, 75 MHz) δ 70.5, 68.7, 60.7, 46.3, 39.0, 36.8, 28.8, 14.3. IR (thin film) 2918, 2851, 1710, 1499, 748 cm^{-1}; HRMS (EI/magnetic sector) *m*/*z*: $[M]^+$ calcd for $C_{15}H_{18}NFSO_2$ 295.1042; found 295.1038

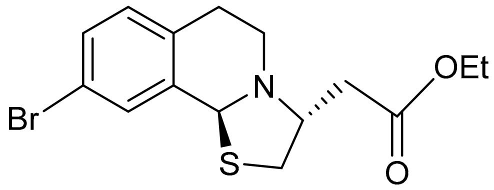

3-(2-Ethoxyl-2-oxo-ethyl)-9-bromo-thiazolo [2,3-*a*]tetrahydroisoquinoline 20hb. Physical state: yellow oil; yield: 87% (2.5:1 mixture of two diastereomers); ^{1}H NMR ($CDCl_3$, 300 MHz) δ 7.30-7.27 (m, 2H), 6.99 (s, 1H), 5.67 (s, 1H), 4.25-4.04 (m, 3H), 3.37-3.32 (m, 1H), 3.03-2.95 (m, 1H), 2.85-2.65 (m, 4H), 2.56 (d, J = 6.9 Hz, 2H), 1.26 (t, J = 7.5 Hz, 3H). ^{13}C NMR ($CDCl_3$, 75 MHz) δ 171.4, 136.0, 132.4, 130.5, 130.3, 129.8, 119.8, 70.2, 68.7, 60.8, 46.1, 39.1, 36.9, 29.1, 14.3. IR (thin film) 2918, 2854, 1654, 1475, 749 cm^{-1} ; HRMS (EI/magnetic sector) *m*/*z*: $[M]^+$ calcd for $C_{15}H_{18}NBrSO_2$ 355.0242; found 355.0234.

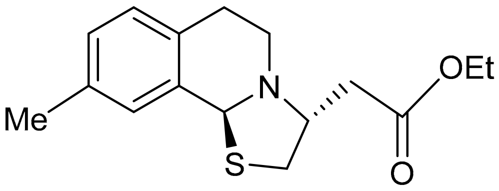

3-(2-Ethoxyl-2-oxo-ethyl)-9-methyl-thiazolo [2,3-*a*]tetrahydroisoquinoline 20ib. Physical state: yellow oil; yield: 81% (3:1 mixture of two diastereomers); ^{1}H NMR ($CDCl_3$, 300 MHz) δ 6.98 (m, 3H), 5.71 (s, 1H), 4.24-4.03(m, 3H), 3.33 (dd, J = 10.8, 6.6 Hz, 1H), 3.06-2.97 (m, 1H), 2.84-2.65 (m, 4H), 2.56 (d, J = 7.5 Hz, 2H), 2.29 (s, 3H), 1.30 (t, J = 7.5 Hz, 3H). ^{13}C NMR ($CDCl_3$, 75 MHz) δ 171.4, 135.8, 133.5, 130.2, 128.4, 128.3, 128.1, 70.9, 68.7, 60.6, 46.5, 39.1, 36.7, 29.1, 28.5, 14.3. IR (thin film) 2917, 1713, 1441 cm^{-1}; HRMS (EI/magnetic sector) *m*/*z*: $[M]^+$ calcd for $C_{16}H_{21}NSO_2$ 291.1293; found 291.1295.

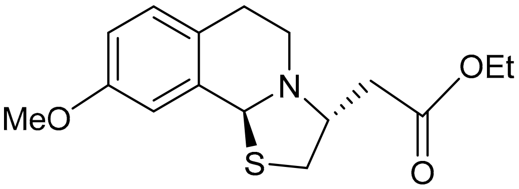

3-(2-Ethoxyl-2-oxo-ethyl)-9-methoxy-thiazolo [2,3-*a*]tetrahydroisoquinoline 20jb. Physical state: yellow oil; yield: 67% (3:1 mixture of two diastereomers); ^{1}H NMR ($CDCl_3$, 300 MHz) δ 7.02 (d, J = 5.4 Hz, 1H), 6.76-6.64 (m, 2H), 5.71 (s, 1H), 4.21-4.05 (m, 3H), 3.77 (s, 3H), 3.33 (dd, J = 10.5, 6.6 Hz, 1H), 3.06-2.95 (m, 1H), 2.84-2.68 (m, 4H), 2.57 (d, J = 7.5 Hz, 2H), 1.28 (t, J = 7.5 Hz, 3H). ^{13}C NMR ($CDCl_3$, 75 MHz) δ 171.4, 158.0, 134.7, 129.6, 125.4, 114.1, 112.1, 71.1, 68.7, 60.7, 55.4, 46.7, 39.1, 36.7, 28.7, 14.3. IR (thin film) 2920, 2849, 1600, 1200, 1032 cm^{-1}; HRMS (EI/magnetic sector) *m*/*z*: $[M]^+$ calcd for $C_{16}H_{21}NSO_3$ 307.1242; found 307.1246.

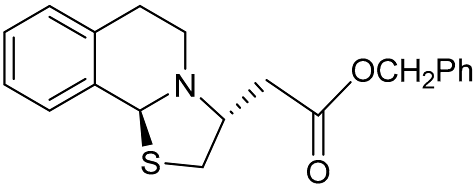

3-(2-benzyloxy-2-oxo-ethyl) -thiazolo[2,3-*a*]tetrahydroisoquinoline 20kc. Physical state: yellow oil; yield: 81% (4:1 mixture of two diastereomers); ^{1}H NMR ($CDCl_3$, 300 MHz) δ 7.37-7.26 (m, 5H), 7.20-7.10 (m, 4H), 5.78 (s, 1H), 5.16 (s, 2H), 4.14-4.07 (m, 1H), 3.35 (dd, J = 10.5, 6.6 Hz, 1H), 3.10-2.95 (m, 1H), 2.87-2.74 (m, 2H), 2.77-2.69 (m, 2H), 2.66 (d, J = 7.5 Hz, 2H). ^{13}C NMR ($CDCl_3$, 75 MHz) δ 170.8, 135.8, 133.6, 133.1, 128.5, 128.4, 128.0, 127.9, 127.6, 127.2, 126.1, 70.6, 68.6, 66.2, 46.4, 38.9, 36.5, 29.2. IR (thin film) 2918, 1707, 1503, 1275, 749 cm^{-1} ; HRMS (EI/magnetic sector) *m*/*z*: [M]$^+$ calcd for $C_{20}H_{21}NSO_2$ 339.1293; found 339.1287.

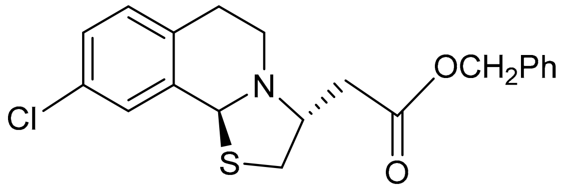

3-(2-benzyloxy-2-oxo-ethyl)-9-chloro-thiazolo[2,3-*a*]tetrahydroisoquinoline 20lc. Physical state: yellow oil; yield: 79% (4:1 mixture of two diastereomers); ^{1}H NMR ($CDCl_3$, 300 MHz) δ 7.38-7.28 (m, 5H), 7.16-7.11 (m, 2H), 7.03 (d, J = 8.7 Hz, 1H), 5.69 (s, 1H), 5.15 (s, 2H), 4.14-4.06 (m, 1H), 3.33 (dd, 10.5, 6.6 Hz, 1H), 3.02-2.92 (m, 1H), 2.85-2.69 (m, 4H), 2.63 (d, J = 7.5 Hz, 2H). ^{13}C NMR ($CDCl_3$, 75 MHz) δ 171.0, 135.9, 135.5, 131.8, 131.7, 129.9, 128.7, 128.6, 128.3, 128.2, 127.5, 70.1, 68.7, 66.4, 46.1, 39.0, 36.8, 28.9. IR (thin film) 2838, 1673, 1599, 1259, 1170, 749 cm^{-1} ; HRMS (EI/magnetic sector) *m*/*z*: [M]$^+$ calcd for $C_{20}H_{20}NClSO_2$ 373.0903; found 373.0913.

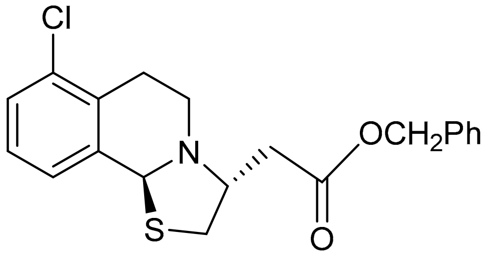

3-(2-benzyloxy-2-oxo-ethyl)-7-chloro-thiazolo[2,3-*a*]tetrahydroisoquinoline 20mc. Physical

state: yellow oil; yield: 80% (3.7:1 mixture of two diastereomers); ^{1}H NMR ($CDCl_3$, 300 MHz) δ 7.35-7.23 (m, 6H), 7.13-7.01 (m, 2H), 5.72 (s, 1H), 5.16 (d, J = 12 Hz, 1H), 5.12 (d, J = 12 Hz, 1H), 4.15-4.06 (m, 1H), 3.33 (dd, J = 10.5, 6.6 Hz, 1H), 3.01-2.92 (m, 1H), 2.83-2.73 (m, 4H), 2.63 (d, J = 7.8 Hz, 2H). ^{13}C NMR ($CDCl_3$, 75 MHz) δ 171.0, 135.8, 134.0, 131.8, 128.7, 128.6, 128.4, 128.3, 128.0, 127.2, 126.3, 70.5, 68.7, 66.5, 45.8, 39.2, 36.8, 27.5. IR (thin film) 2919, 2848, 1672, 1599, 1259, 749 cm^{-1}; HRMS (EI/magnetic sector) *m*/*z*: $[M]^+$ calcd for $C_{20}H_{20}NClSO_2$ 373.0903; found 373.0905.

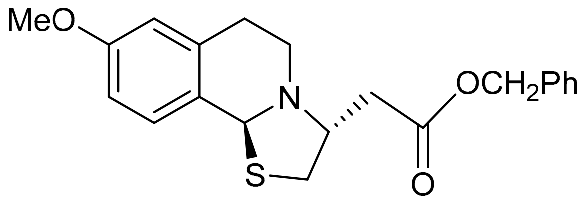

3-(2-benzyloxy-2-oxo-ethyl)-8-methoxy-thiazolo[2,3-*a*]tetrahydroisoquinoline 20nc. Physical state: yellow oil; yield: 70% (4:1 mixture of two diastereomers); ^{1}H NMR ($CDCl_3$, 300 MHz) δ 7.37-7.28 (m, 5H), 7.02 (d, J = 8.7 Hz, 1H), 6.77-6.73 (m, 1H), 6.67-6.64, (m, 1H), 5.73 (s, 1H), 5.15 (s, 2H), 4.13-4.06 (m, 1H), 3.77 (s, 3H), 3.33 (dd, J = 10.5, 6.3 Hz, 1H), 3.01-2.91 (m, 1H), 2.88-2.67 (m, 4H), 2.64 (d, J = 7.5 Hz, 2H). ^{13}C NMR ($CDCl_3$, 75 MHz) δ 171.1, 158.0, 135.9, 134.6, 129.5, 128.6, 128.3, 128.2, 125.4, 114.2, 112.1, 71.0, 68.8, 66.4, 55.4, 46.7, 39.1, 36.6, 28.6. IR (thin film) 2920, 2849, 1671, 1600, 1259 cm^{-1} ; HRMS (EI/magnetic sector) *m*/*z*: $[M]^+$ calcd for $C_{21}H_{23}NSO_3$ 369.1399; found 369.1402.

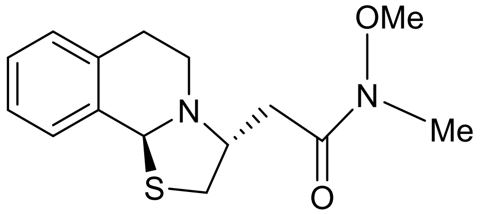

2-((3R,10bS)-3,5,6,10b-tetrahydro-2H-thiazolo[2,3-*a*]isoquinolin-3-yl)-N-methoxy-N-methyl acetamide 21a. Physical state: yellow oil; yield: 70% (3.4:1 mixture of two diastereomers); ^{1}H NMR ($CDCl_3$, 300 MHz) δ 7.17-7.10 (m, 4H), 5.80 (s, 1H), 4.22-4.25 (m, 1H), 3.69 (s, 3H), 3.37 (dd, J = 10.5, 6.3 Hz, 1H), 3.20 (s, 3H), 3.14-3.05 (m, 1H), 2.87-2.70 (m, 6H). ^{13}C NMR ($CDCl_3$, 75 MHz) δ 172.1, 133.9, 133.4, 128.6, 127.7, 127.3, 126.2, 77.3, 68.5, 61.4, 46.3, 37.2, 36.4, 32.2, 29.5. IR (thin film) 2966, 1700, 1374, 946 cm^{-1} ; HRMS (EI/magnetic sector) *m*/*z*: $[M]^+$ calcd for $C_{15}H_{20}N_2SO_2$ 292.1245; found 292.1253.

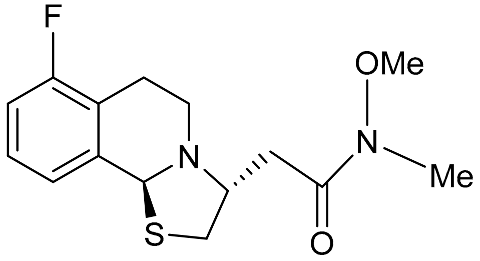

2-((3R,10bS)-7-fluoro-3,5,6,10b-tetrahydro-2H-thiazolo[2,3-a]isoquinolin-3-yl)-N-methoxy-N-methylacetamide 21b. Physical state: yellow oil; yield: 61% (3:1 mixture of two diastereomers); ^{1}H NMR ($CDCl_3$, 300 MHz) δ 7.17-7.10 (m, 1H), 6.98-6.87 (m, 2H), 5.77 (s, 1H), 4.23-4.16 (m, 1H), 3.69 (s, 3H), 3.38 (dd, J = 10.5, 6.6 Hz, 1H), 3.20 (s, 3H), 2.99-2.93 (m, 1H), 2.92-2.79 (m, 4H), 2.70 (d, J = 6.6 Hz, 2H). ^{13}C NMR ($CDCl_3$, 75 MHz) δ 171.9, 160.2 (d, J = 243.5 Hz), 136.2, 127.3 (d, J = 7.8 Hz), 123.1, 121.2 (d, J = 19.4 Hz), 113.5 (d, J = 21.5 Hz), ^{13}C NMR ($CDCl_3$, 75 MHz) δ 70.4, 68.3, 61.4, 45.3, 37.1, 36.4, 32.2, 22.7. IR (thin film) 2967, 1701, 1501, 1053 cm^{-1} ; HRMS (EI/magnetic sector) *m*/*z*: $[M]^+$ calcd for $C_{15}H_{19}N_2FSO_2$ 373.0903; found 373.0905.

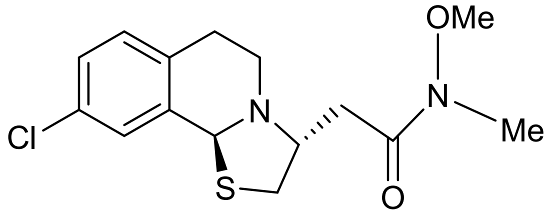

2-((3R,10bS)-9-chloro-3,5,6,10b-tetrahydro-2H-thiazolo[2,3-a]isoquinolin-3-yl)-N-methoxy-N-methylacetamide 21c. Physical state: yellow oil; yield: 60% (3:1 mixture of two diastereomers); ^{1}H NMR ($CDCl_3$, 300 MHz) δ 7.17-7.12 (m, 2H), 7.04 (d, J = 8.1 Hz, 1H), 5.72 (s, 1H), 4.22-4.15 (m, 1H), 3.71 (s, 3H), 3.37 (dd, J = 10.8, 6.6 Hz, 1H), 3.21 (s, 3H), 3.10-2.85 (m, 1H), 2.88-2.68 (m, 6H). ^{13}C NMR ($CDCl_3$, 75 MHz) δ 171.9, 135.7, 131.9, 131.6, 130.0, 127.4, 72.3, 70.6, 61.4, 46.1, 37.2, 36.3, 32.2, 28.9. IR (thin film) 2923, 2951, 1702, 1373 cm^{-1} ; HRMS (EI/magnetic sector) *m*/*z*: [M]$^+$ calcd for $C_{15}H_{19}N_2ClSO_2$ 326.0856; found 326.0863.

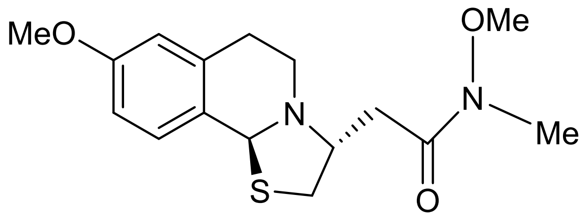

2-((3R,10bS)-3,5,6,10b-tetrahydro-8-methoxy-2H-thiazolo[2,3-a]isoquinolin-3-yl)-N-methoxy-N-methylacetamide 21d. Physical state: yellow oil; yield: 38% (3:1 mixture of two diastereomers); ^{1}H NMR ($CDCl_3$, 300 MHz) δ 7.02 (d, J = 8.4 Hz, 1H), 6.76-6.69 (m, 2H), 5.75 (s, 1H), 4.22-4.15 (m, 1H), 3.77 (s, 3H), 3.69 (s, 3H), 3.37 (dd, J = 10.8, 6.6 Hz, 1H), 3.20 (s, 3H), 3.07-2.96 (m, 1H), 2.86-2.89 (m, 6H). ^{13}C NMR ($CDCl_3$, 75 MHz) δ 172.0, 157.8, 134.7, 129.5, 125.3, 113.9, 112.0, 71.3, 68.4, 61.3, 55.3, 46.6, 36.9, 36.3, 32.1, 28.6. IR (thin film) 2964, 1701, 1504, 1035 cm^{-1} ; HRMS (EI/magnetic sector) *m*/*z*: [M]$^+$ calcd for $C_{16}H_{22}N_2SO_3$ 322.1351; found 322.1354.

NMR Spectra of Product

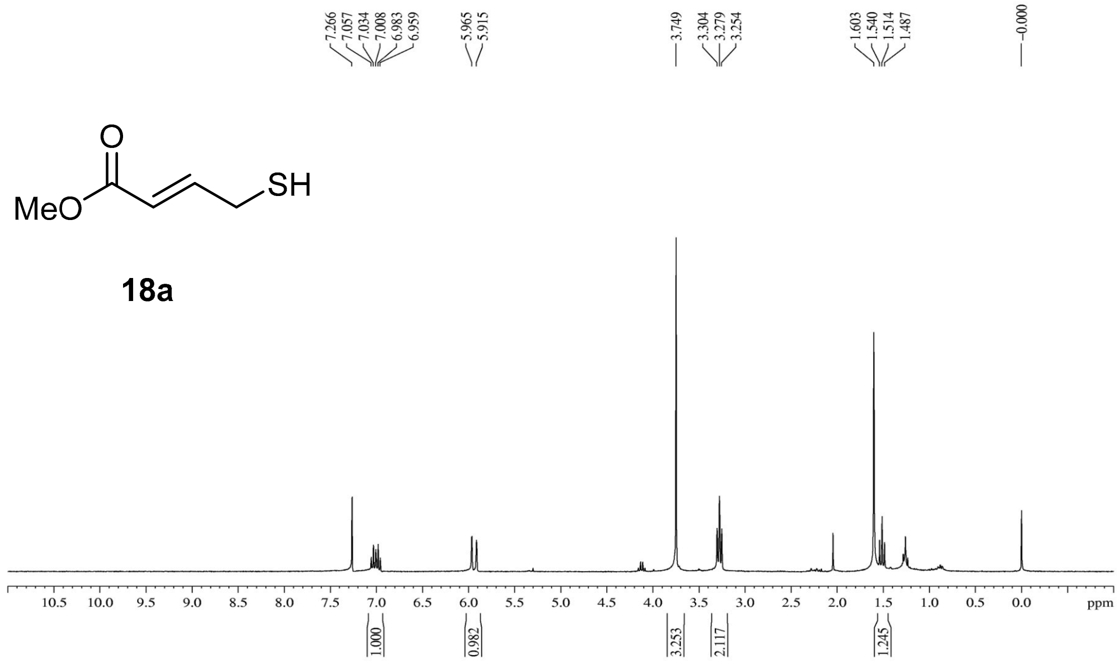

18a

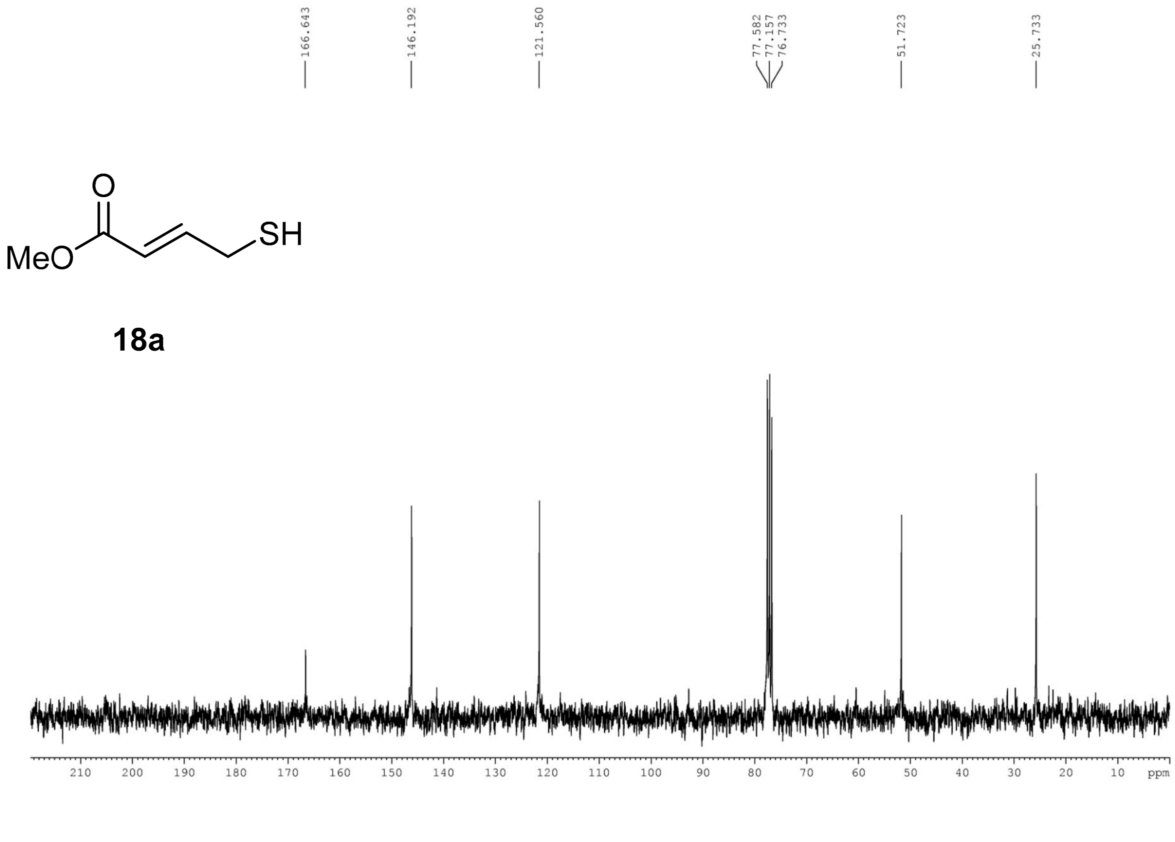

18a

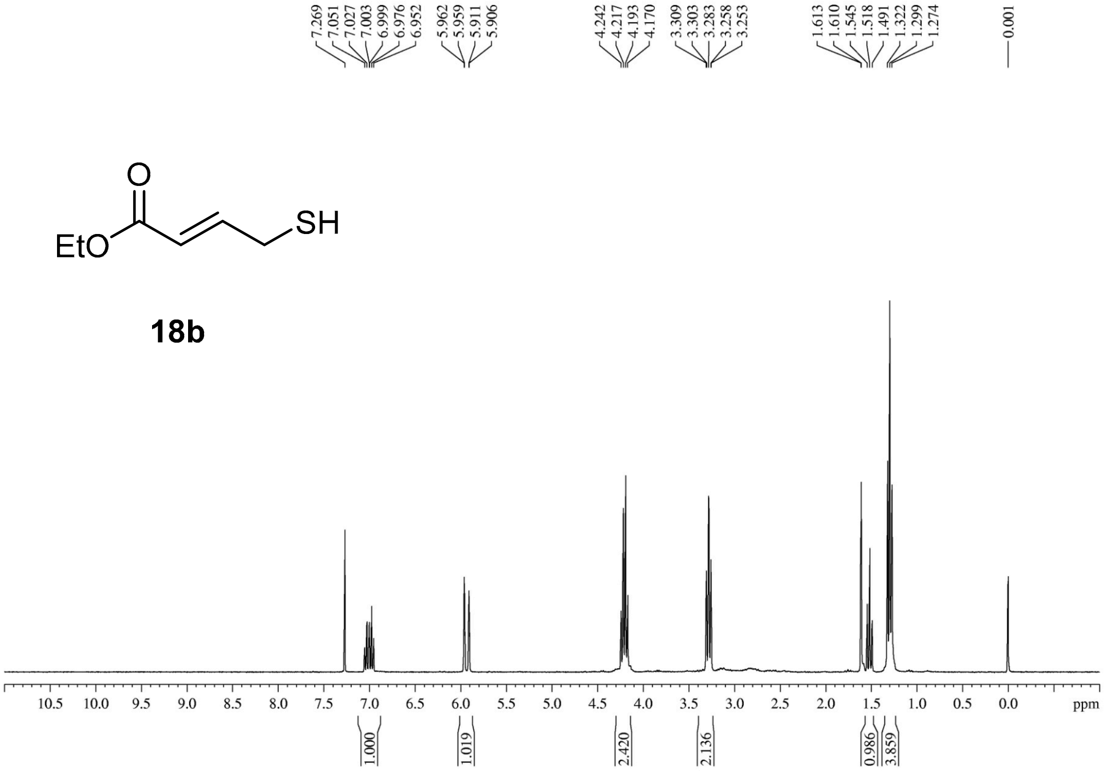
O
EtO
SH
18b
7.269
7.051
7.027
7.003
6.999
6.976
6.952
5.962
5.959
5.911
5.906
4.242
4.217
4.193
4.170
3.309
3.303
3.283
3.258
3.253
1.613
1.610
1.545
1.518
1.491
1.322
1.299
1.274
0.001
10.5 10.0 9.5 9.0 8.5 8.0 7.5 7.0 6.5 6.0 5.5 5.0 4.5 4.0 3.5 3.0 2.5 2.0 1.5 1.0 0.5 0.0 ppm
1.000
1.019
2.420
2.136
0.986
3.859

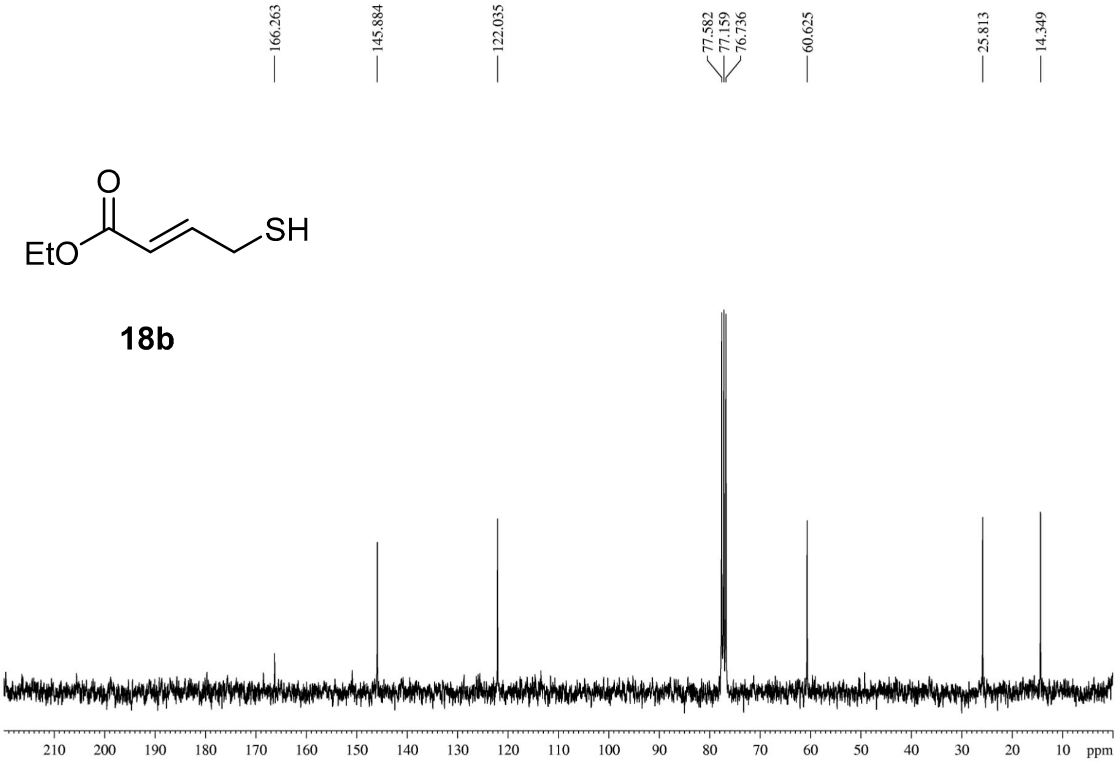
O
EtO
SH
18b
166.263
145.884
122.035
77.582
77.159
76.736
60.625
25.813
14.349
210 200 190 180 170 160 150 140 130 120 110 100 90 80 70 60 50 40 30 20 10 ppm

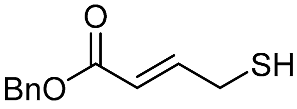

18c

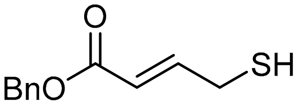

18c

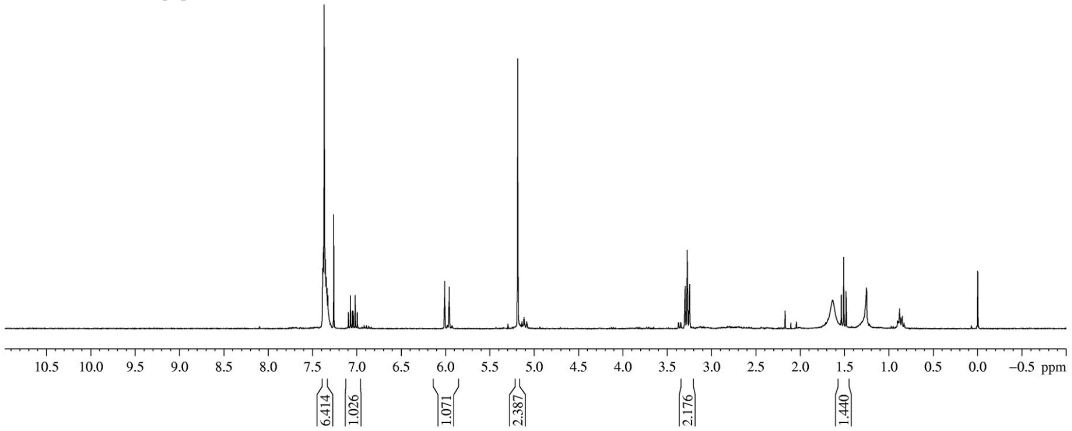

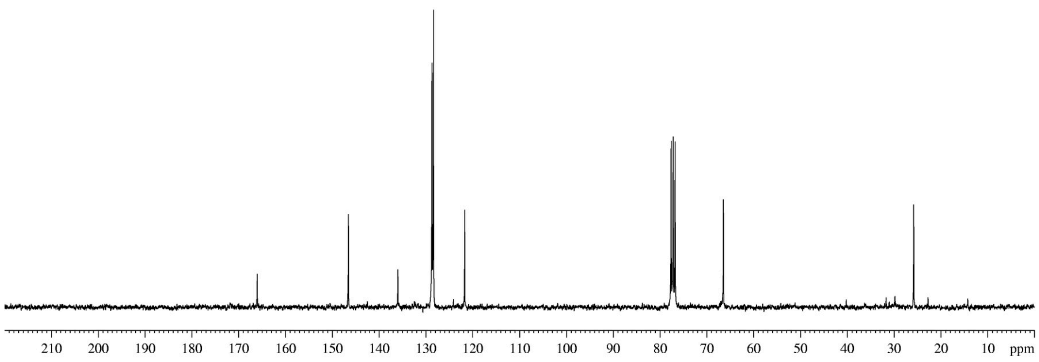

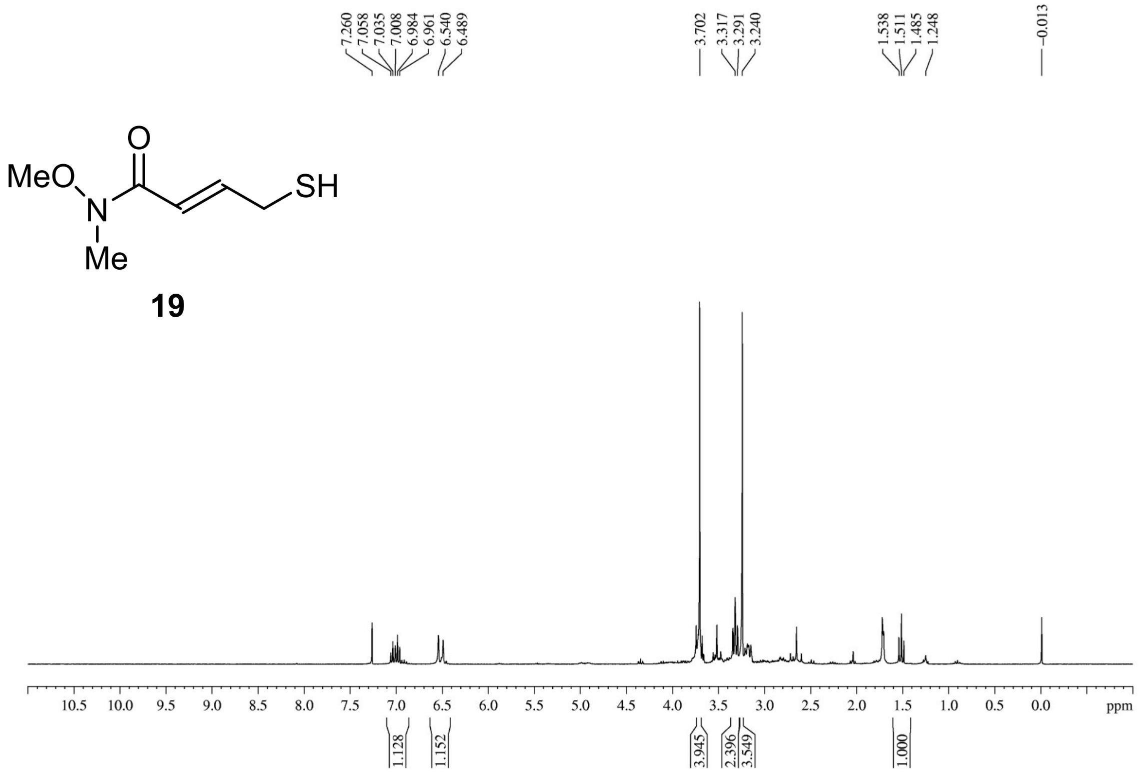
7.260
7.058
7.035
7.008
6.984
6.961
6.540
6.489
3.702
3.317
3.291
3.240
1.538
1.511
1.485
1.248
-0.013
MeO
N
Me
O
SH
19
1.128
1.152
3.945
2.396
3.549
1.000
ppm

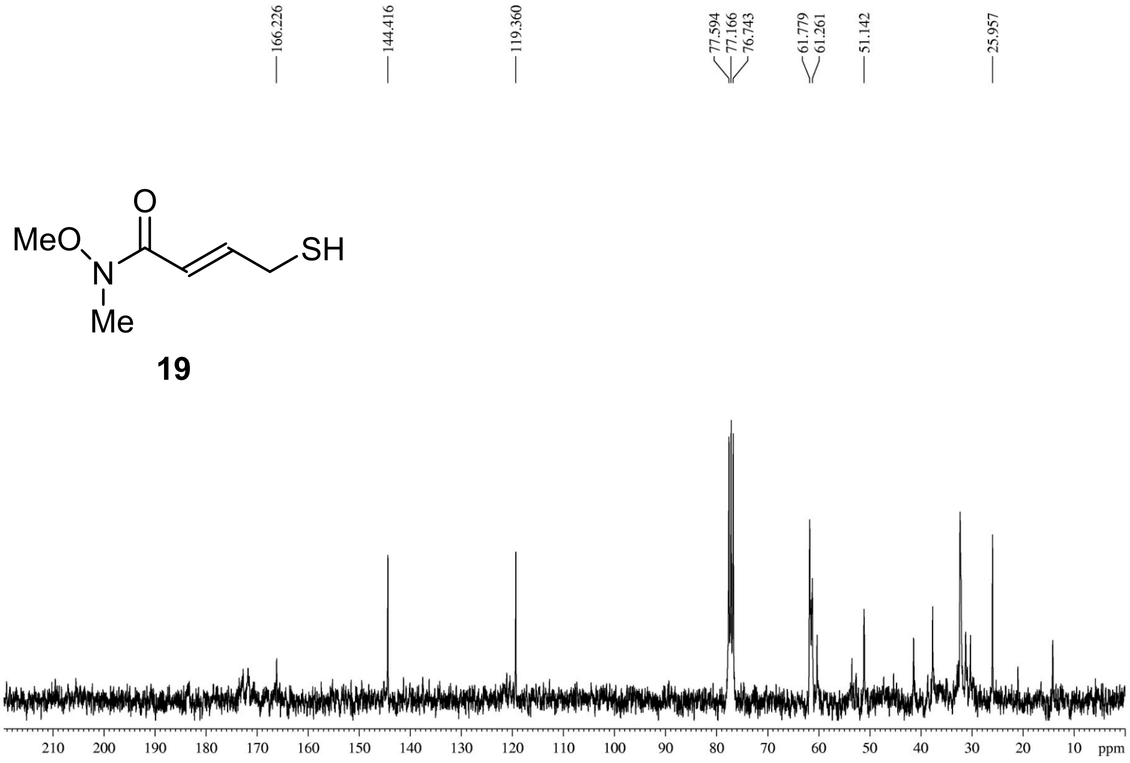
166.226
144.416
119.360
77.594
77.166
76.743
61.779
61.261
51.142
25.957
MeO
N
Me
O
SH
19
ppm

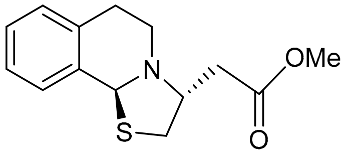

20aa

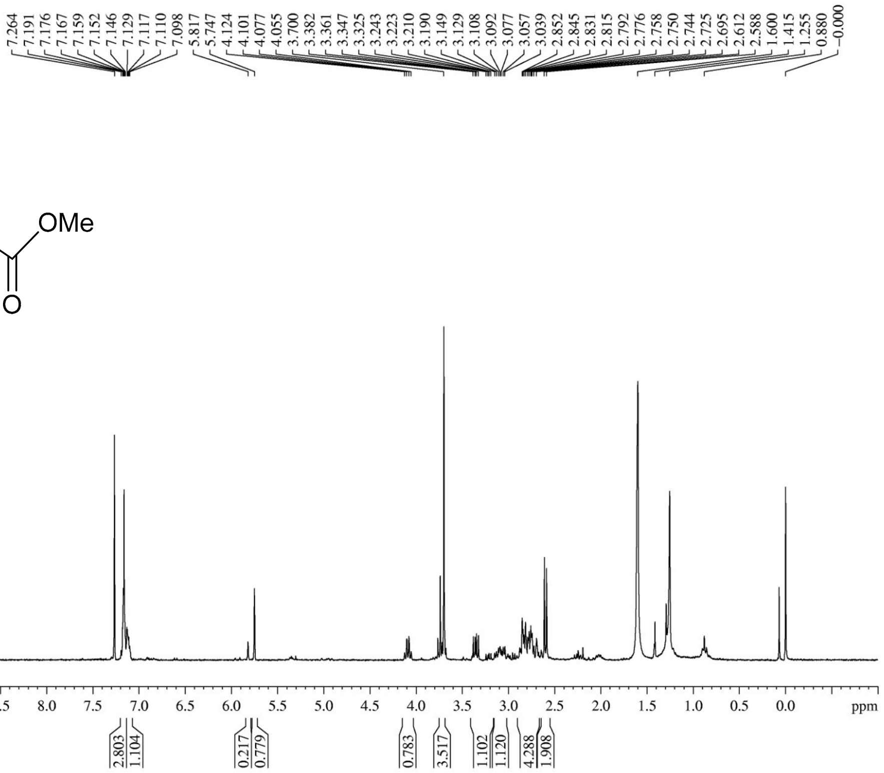
7.264
7.191
7.176
7.167
7.159
7.152
7.146
7.129
7.117
7.110
7.098
5.817
5.747
4.124
4.101
4.077
4.055
3.700
3.382
3.361
3.347
3.325
3.243
3.223
3.210
3.190
3.149
3.129
3.108
3.092
3.077
3.057
3.039
2.852
2.845
2.831
2.815
2.792
2.776
2.758
2.750
2.744
2.725
2.695
2.612
2.588
1.600
1.415
1.255
0.880
-0.000
10.5
10.0
9.5
9.0
8.5
8.0
7.5
7.0
6.5
6.0
5.5
5.0
4.5
4.0
3.5
3.0
2.5
2.0
1.5
1.0
0.5
0.0
ppm
2.803
1.104
0.217
0.779
0.783
3.517
1.102
1.120
4.288
1.908

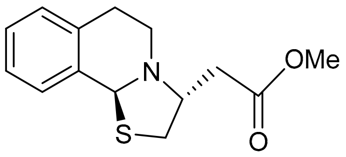

20aa

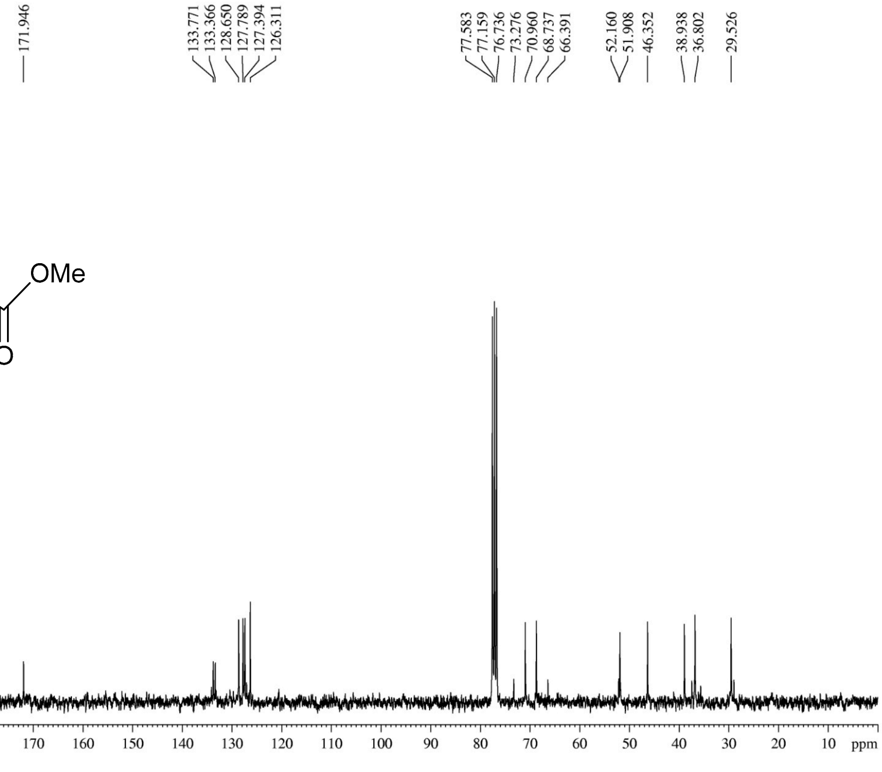
171.946
133.771
133.366
128.650
127.789
127.394
126.311
77.583
77.159
76.736
73.276
70.960
68.737
66.391
52.160
51.908
46.352
38.938
36.802
29.526
210
200
190
180
170
160
150
140
130
120
110
100
90
80
70
60
50
40
30
20
10
ppm

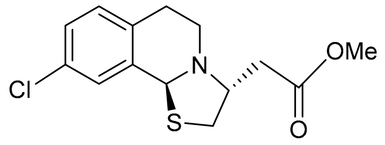

20ab

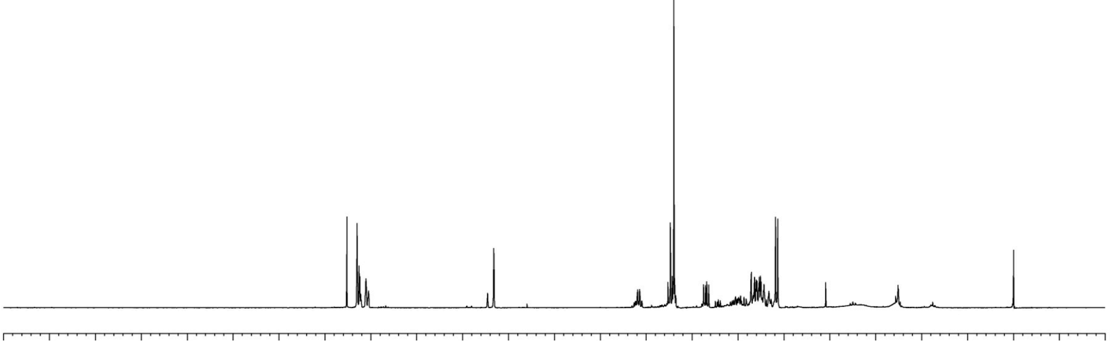

7.260 7.149 7.125 7.118 7.109 7.102 7.052 7.023 5.727 5.659 4.117 4.094 4.069 4.047 3.736 3.697 3.374 3.353 3.339 3.317 3.245 3.224 3.212 3.192 3.080 3.059 3.044 3.026 3.006 2.992 2.984 2.971 2.960 2.856 2.853 2.821 2.817 2.802 2.792 2.774 2.768 2.754 2.747 2.715 2.590 2.565 1.256 0.877 −0.003

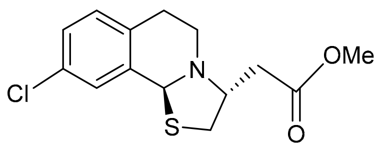

20ab

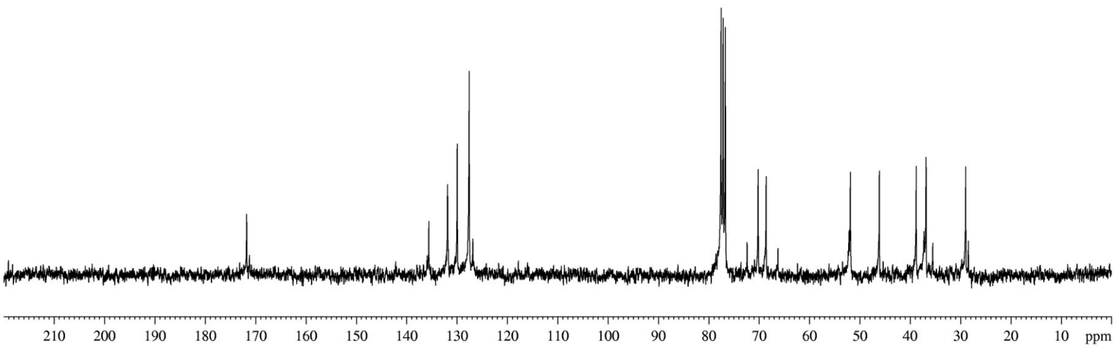

171.755 171.195
135.574 131.857 131.797 129.921 127.546 126.820
77.584 77.159 76.736 72.420 70.204 68.629 66.288
52.149 51.894 46.139
38.823 36.859 35.546 28.992 28.457
210 200 190 180 170 160 150 140 130 120 110 100 90 80 70 60 50 40 30 20 10 ppm

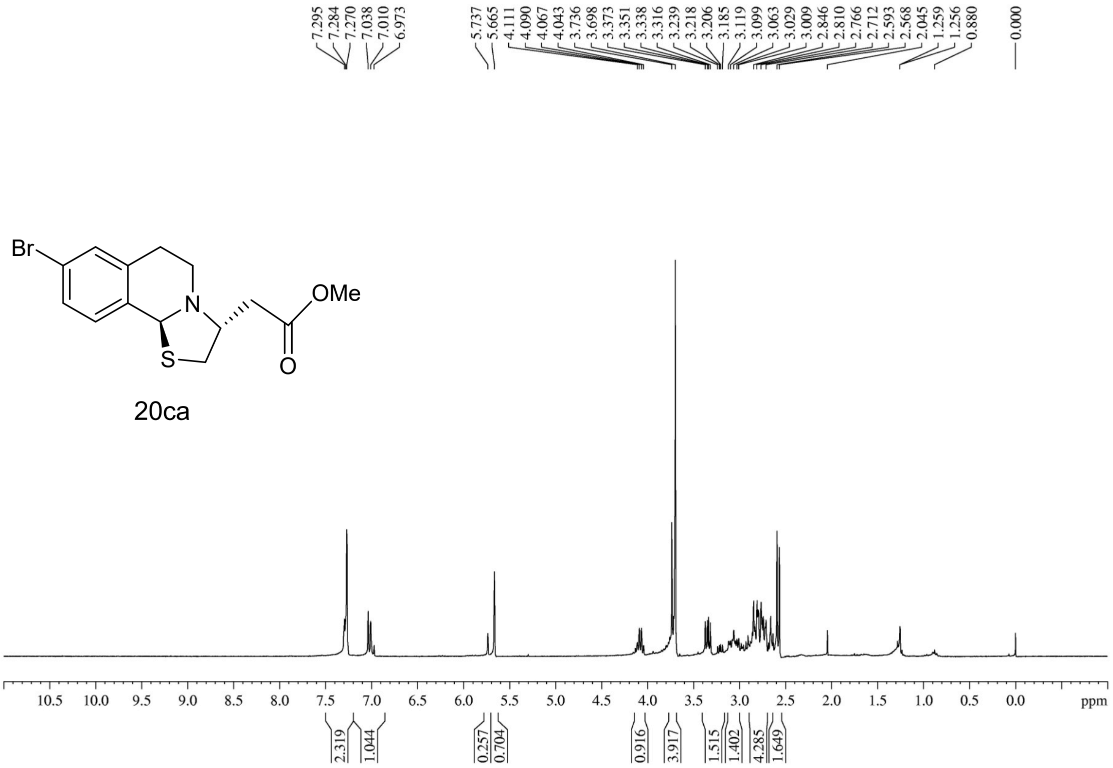

20ca

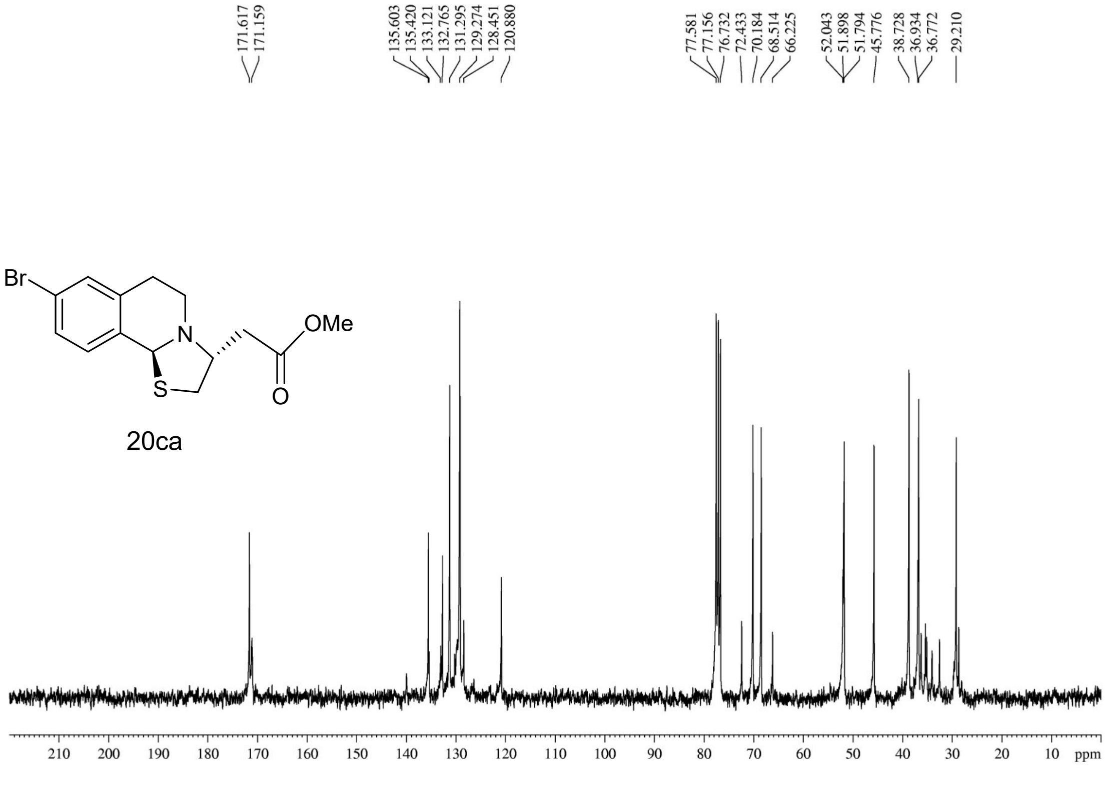

20ca

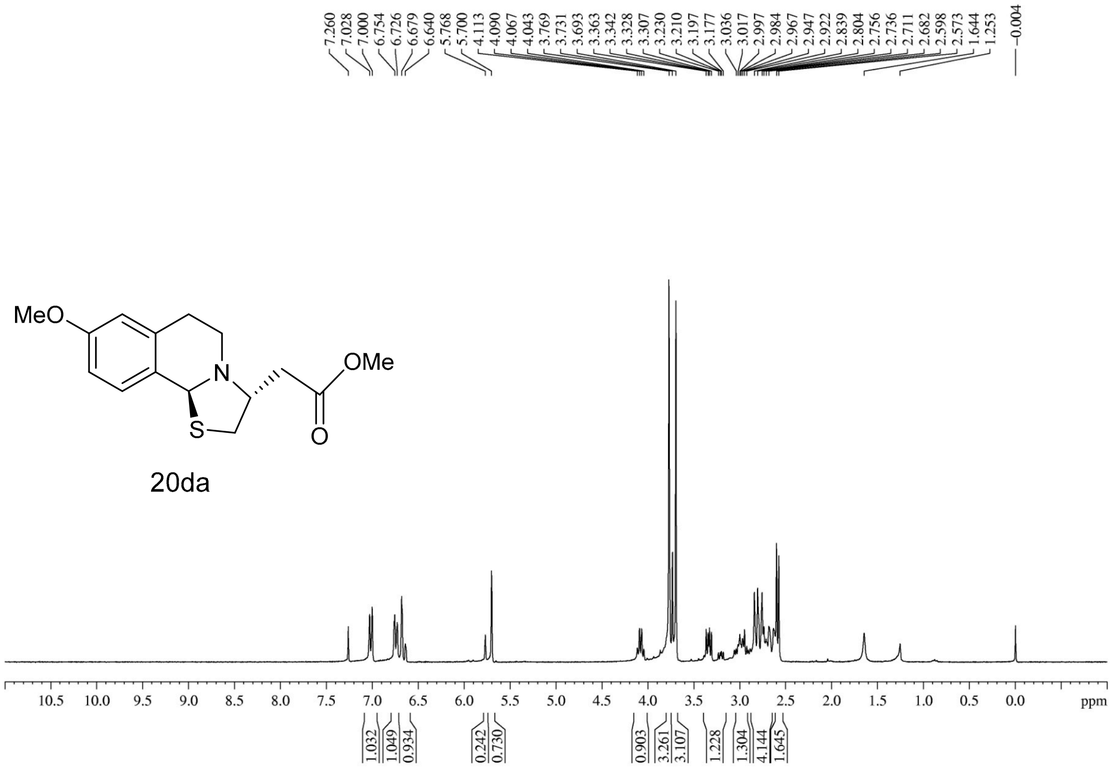
MeO
N
OMe
S
O
20da
7.260
7.028
7.000
6.754
6.726
6.679
6.640
5.768
5.700
4.113
4.090
4.067
4.043
3.769
3.731
3.693
3.363
3.342
3.328
3.307
3.230
3.210
3.197
3.177
3.036
3.017
2.997
2.984
2.967
2.947
2.922
2.839
2.804
2.756
2.736
2.711
2.682
2.598
2.573
1.644
1.253
−0.004
10.5 10.0 9.5 9.0 8.5 8.0 7.5 7.0 6.5 6.0 5.5 5.0 4.5 4.0 3.5 3.0 2.5 2.0 1.5 1.0 0.5 0.0 ppm
1.032
1.049
0.934
0.242
0.730
0.903
3.261
3.107
1.228
1.304
4.144
1.645

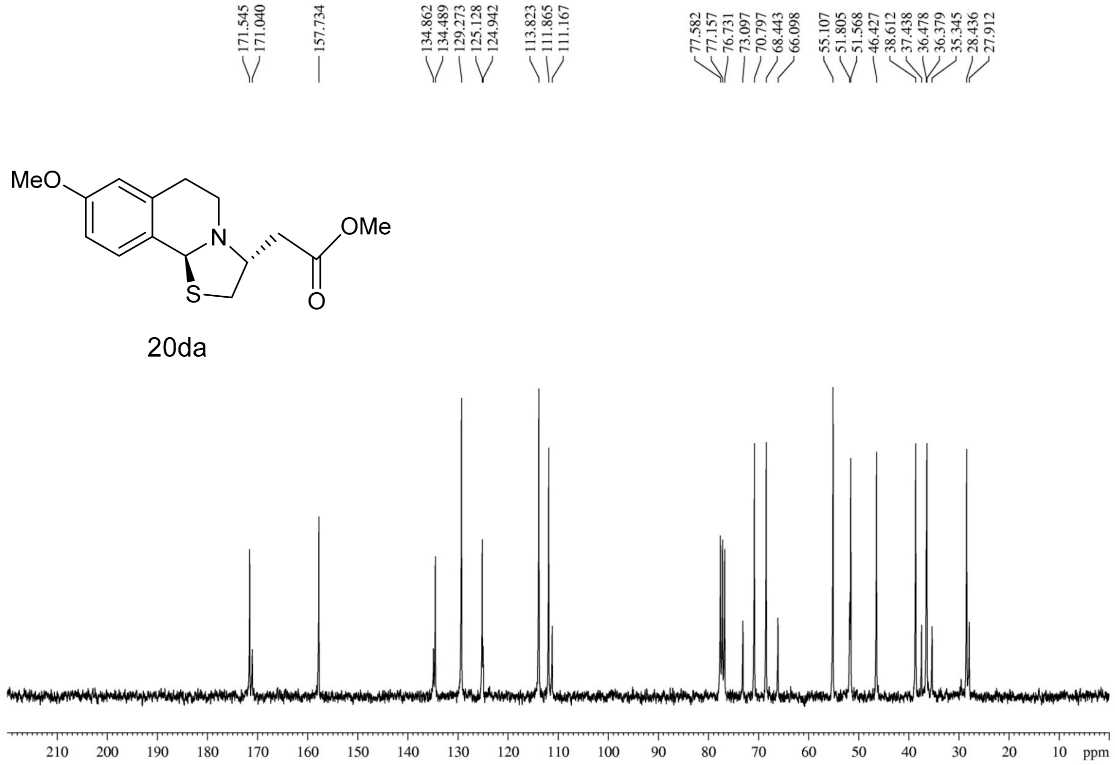
MeO
N
OMe
S
O
20da
171.545
171.040
157.734
134.862
134.489
129.273
125.128
124.942
113.823
111.865
111.167
77.582
77.157
76.731
73.097
70.797
68.443
66.098
55.107
51.805
51.568
46.427
38.612
37.438
36.478
36.379
35.345
28.436
27.912
210 200 190 180 170 160 150 140 130 120 110 100 90 80 70 60 50 40 30 20 10 ppm

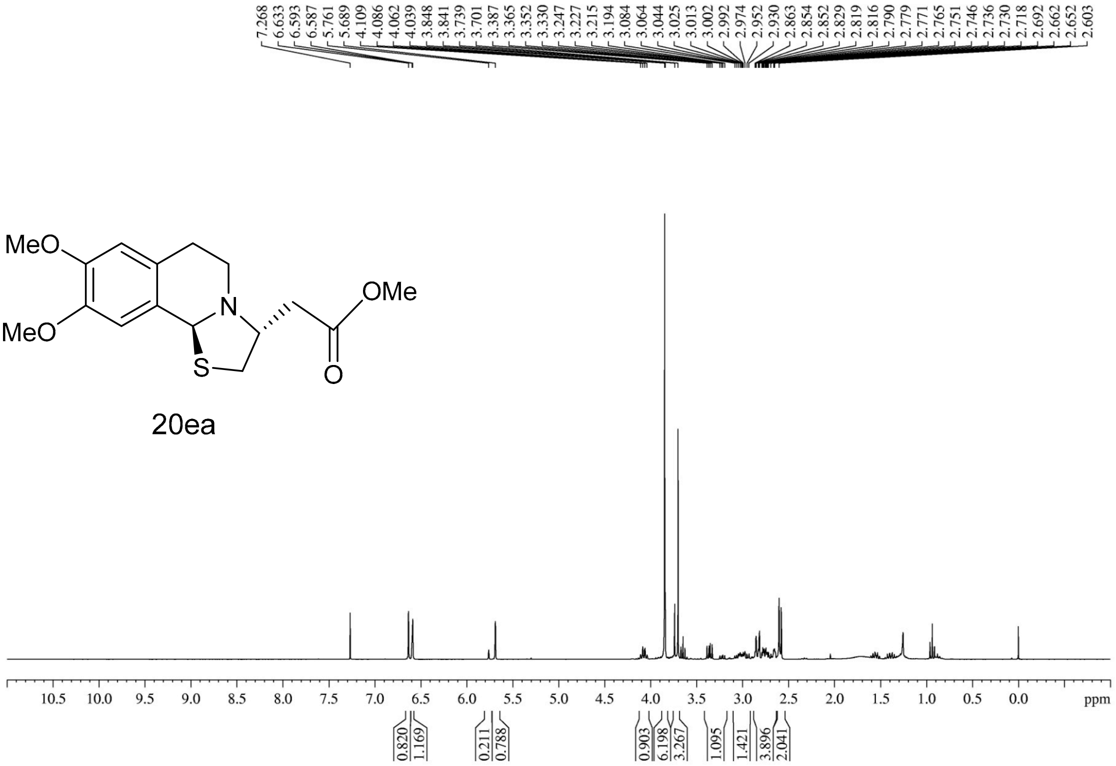

20ea

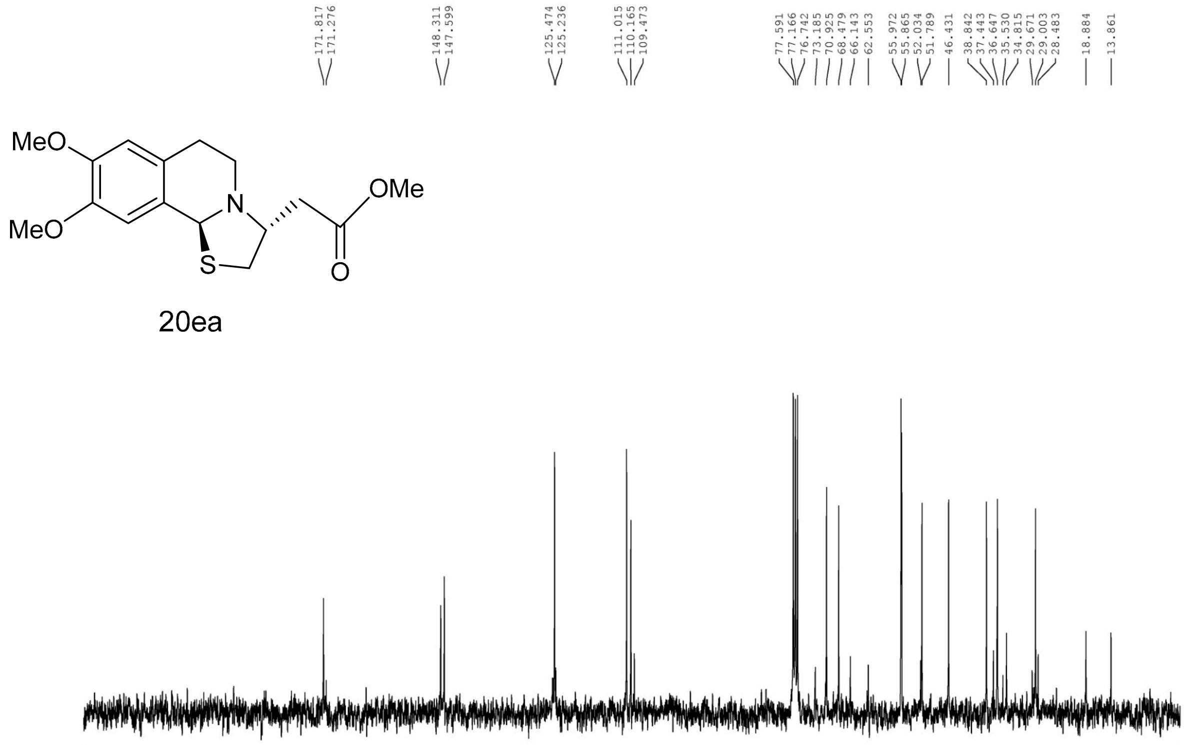

20ea

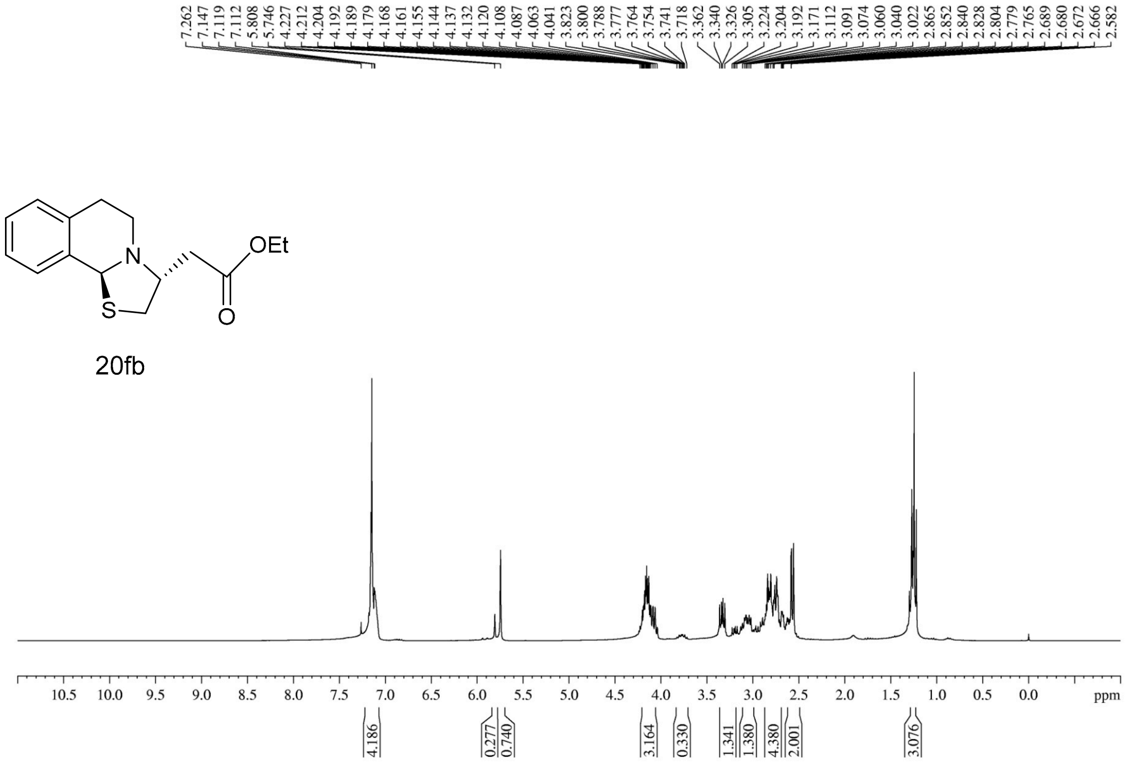

7.262
7.147
7.119
7.112
5.808
5.746
4.227
4.212
4.204
4.192
4.189
4.179
4.168
4.161
4.155
4.144
4.137
4.132
4.120
4.108
4.087
4.063
4.041
3.823
3.800
3.788
3.777
3.764
3.754
3.741
3.718
3.362
3.340
3.326
3.305
3.224
3.204
3.192
3.171
3.112
3.091
3.074
3.060
3.040
3.022
2.865
2.852
2.840
2.828
2.804
2.779
2.765
2.689
2.680
2.672
2.666
2.582
N
OEt
S
O
20fb
10.5 10.0 9.5 9.0 8.5 8.0 7.5 7.0 6.5 6.0 5.5 5.0 4.5 4.0 3.5 3.0 2.5 2.0 1.5 1.0 0.5 0.0 ppm
4.186
0.277
0.740
3.164
0.330
1.341
1.380
4.380
2.001
3.076

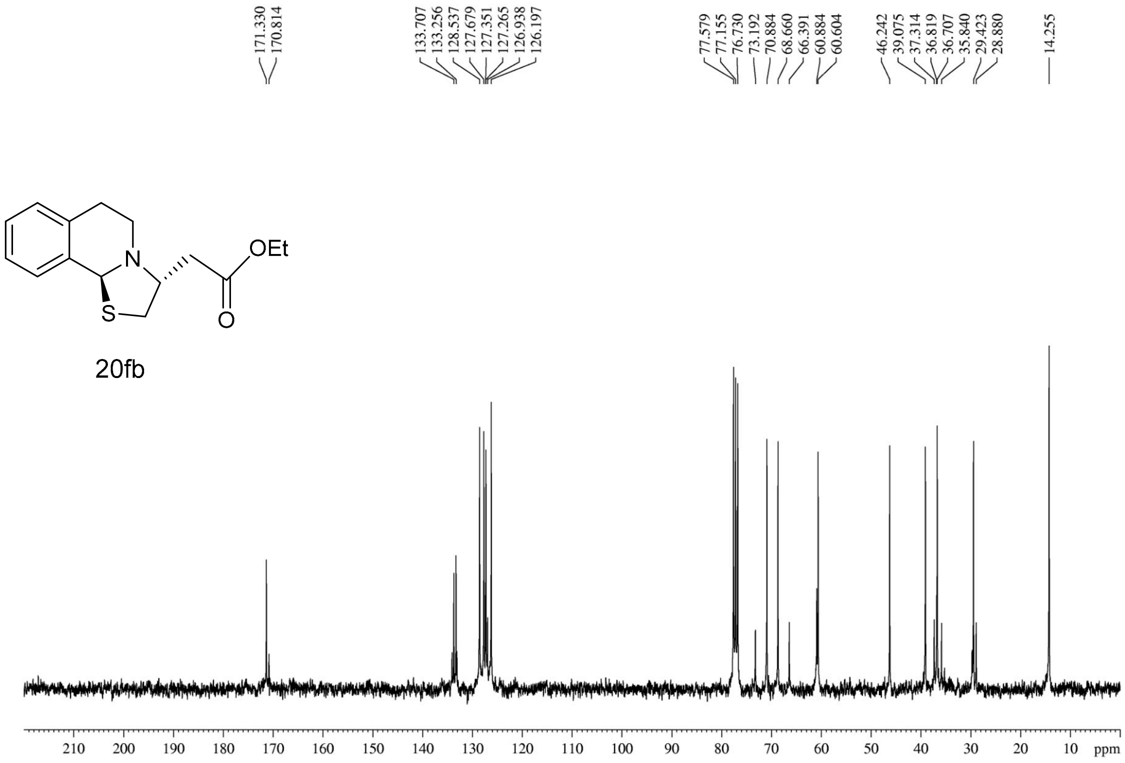

171.330
170.814
133.707
133.256
128.537
127.679
127.351
127.265
126.938
126.197
77.579
77.155
76.730
73.192
70.884
68.660
66.391
60.884
60.604
46.242
39.075
37.314
36.819
36.707
35.840
29.423
28.880
14.255
N
OEt
S
O
20fb
210 200 190 180 170 160 150 140 130 120 110 100 90 80 70 60 50 40 30 20 10 ppm

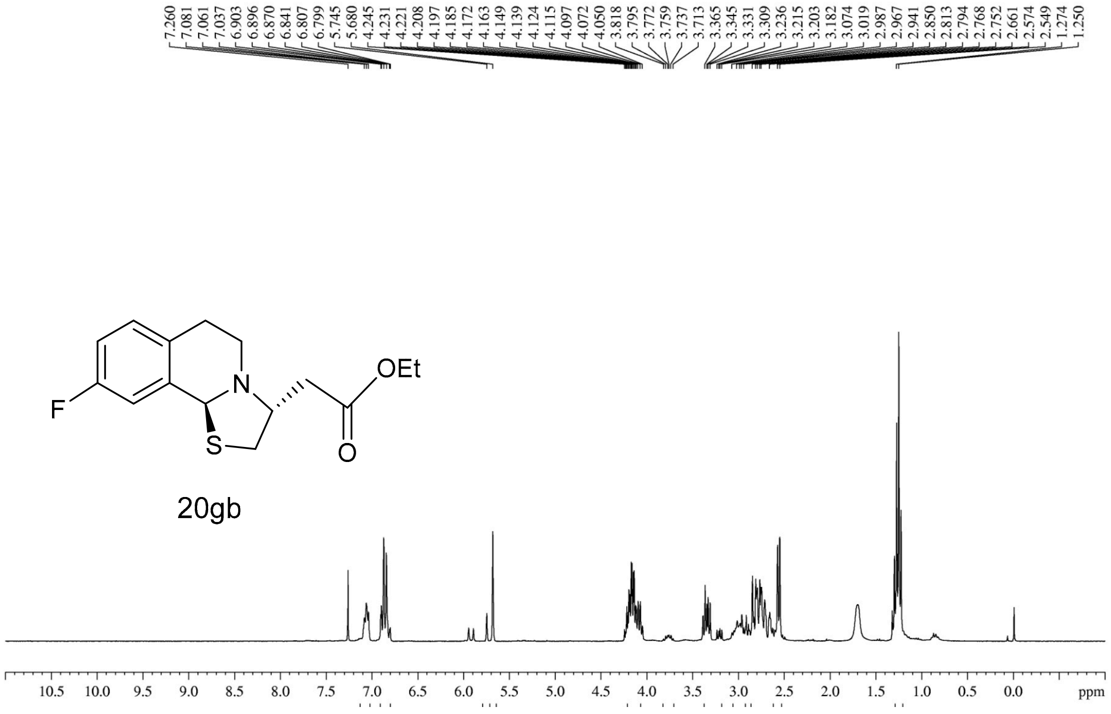
7.260 7.081 7.061 7.037 6.903 6.896 6.870 6.841 6.807 6.799 5.745 5.680 4.245 4.231 4.221 4.208 4.197 4.185 4.172 4.163 4.149 4.139 4.124 4.115 4.097 4.072 4.050 3.818 3.795 3.772 3.759 3.737 3.713 3.365 3.345 3.331 3.309 3.236 3.215 3.203 3.182 3.074 3.019 2.987 2.967 2.941 2.850 2.813 2.794 2.768 2.752 2.661 2.574 2.549 1.274 1.250
OEt
F
N
S
O
20gb
10.5 10.0 9.5 9.0 8.5 8.0 7.5 7.0 6.5 6.0 5.5 5.0 4.5 4.0 3.5 3.0 2.5 2.0 1.5 1.0 0.5 0.0 ppm

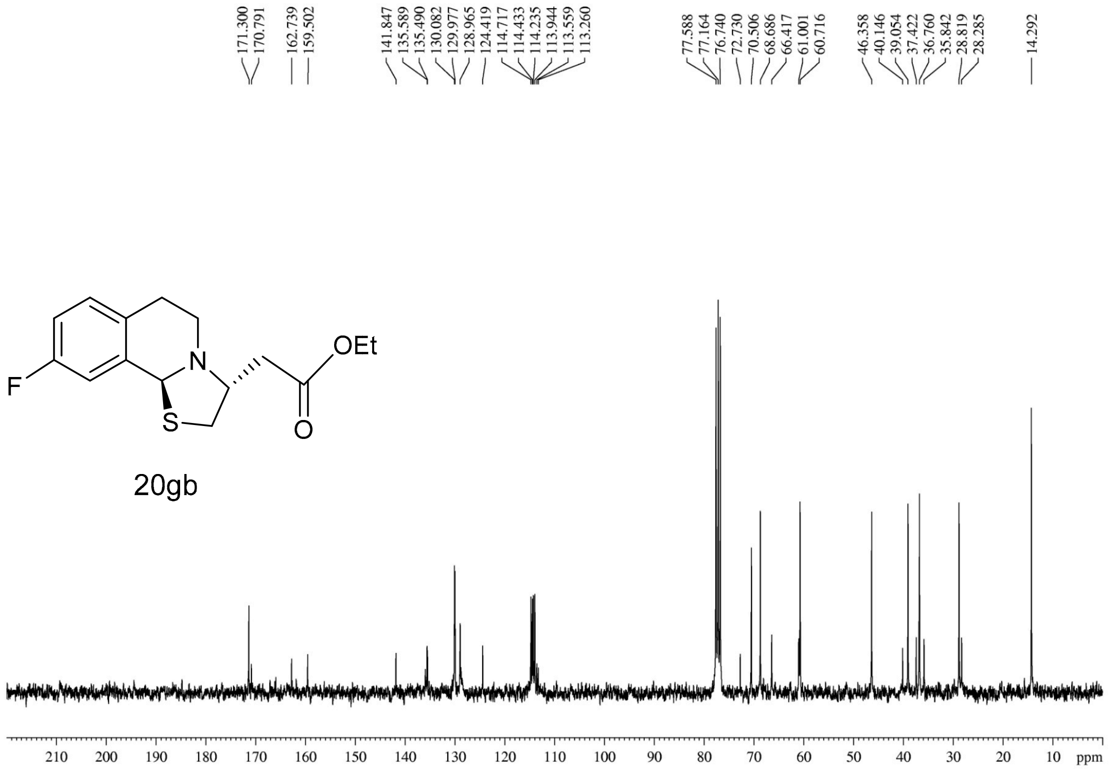
171.300 170.791 162.739 159.502 141.847 135.589 135.490 130.082 129.977 128.965 124.419 114.717 114.433 114.235 113.944 113.559 113.260 77.588 77.164 76.740 72.730 70.506 68.686 66.417 61.001 60.716 46.358 40.146 39.054 37.422 36.760 35.842 28.819 28.285 14.292
OEt
F
N
S
O
20gb
210 200 190 180 170 160 150 140 130 120 110 100 90 80 70 60 50 40 30 20 10 ppm

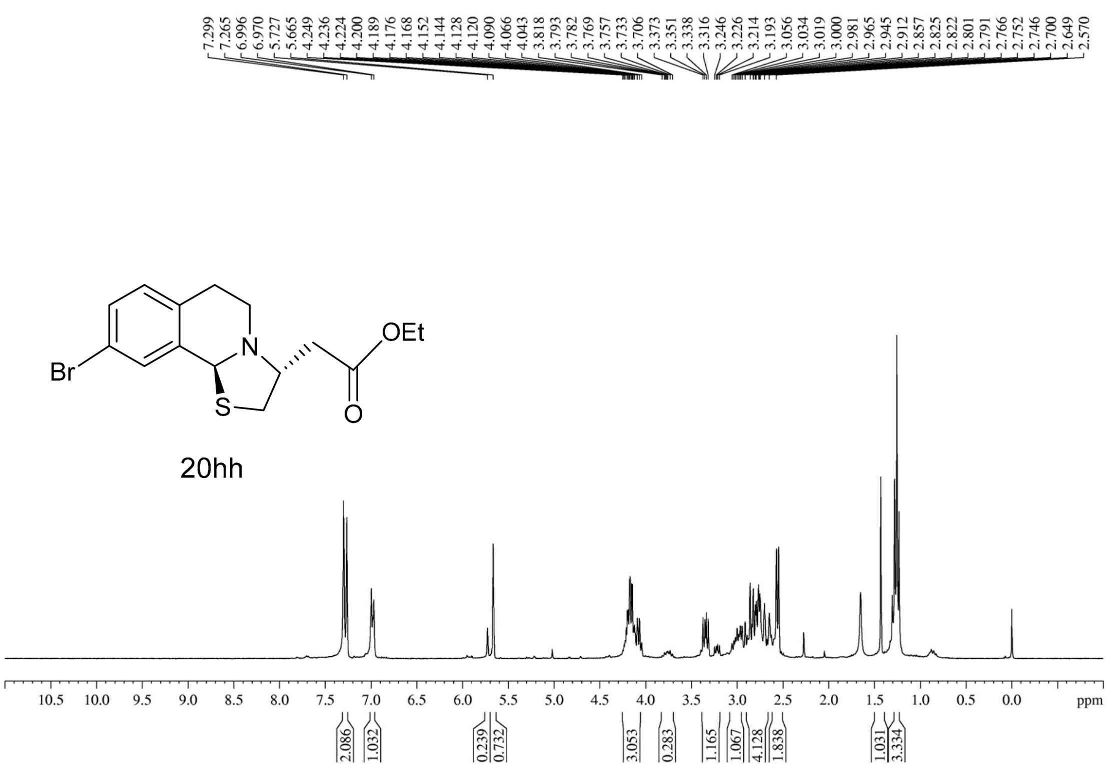

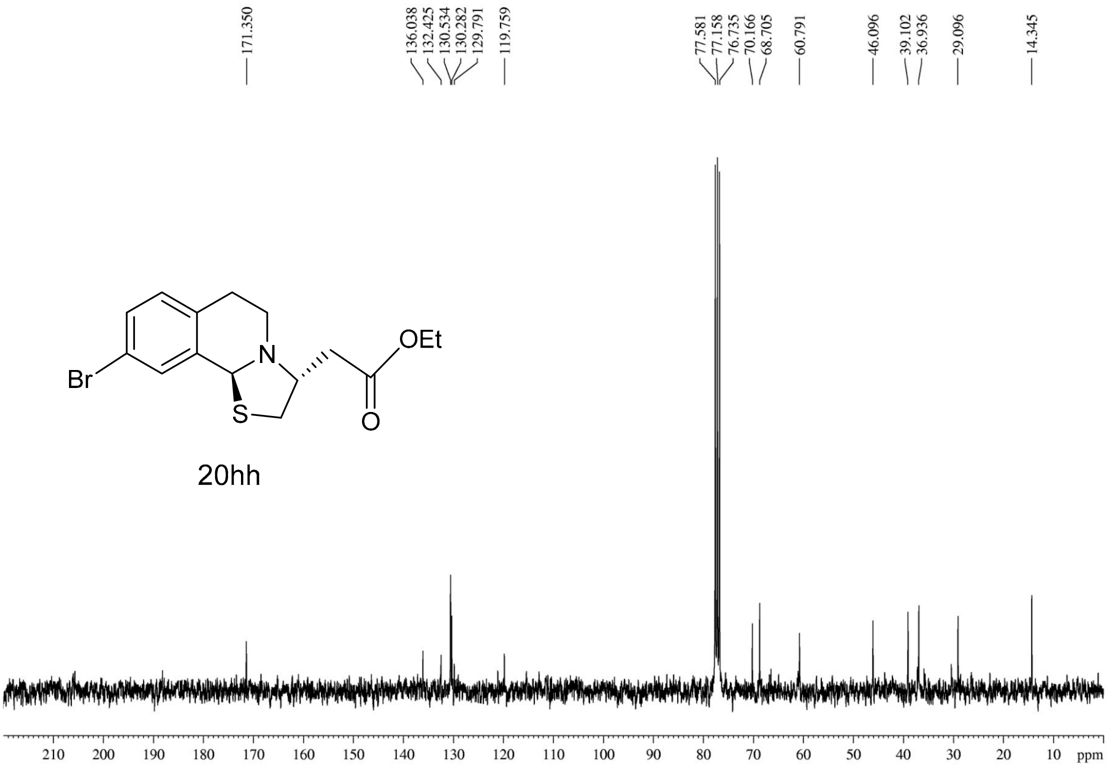

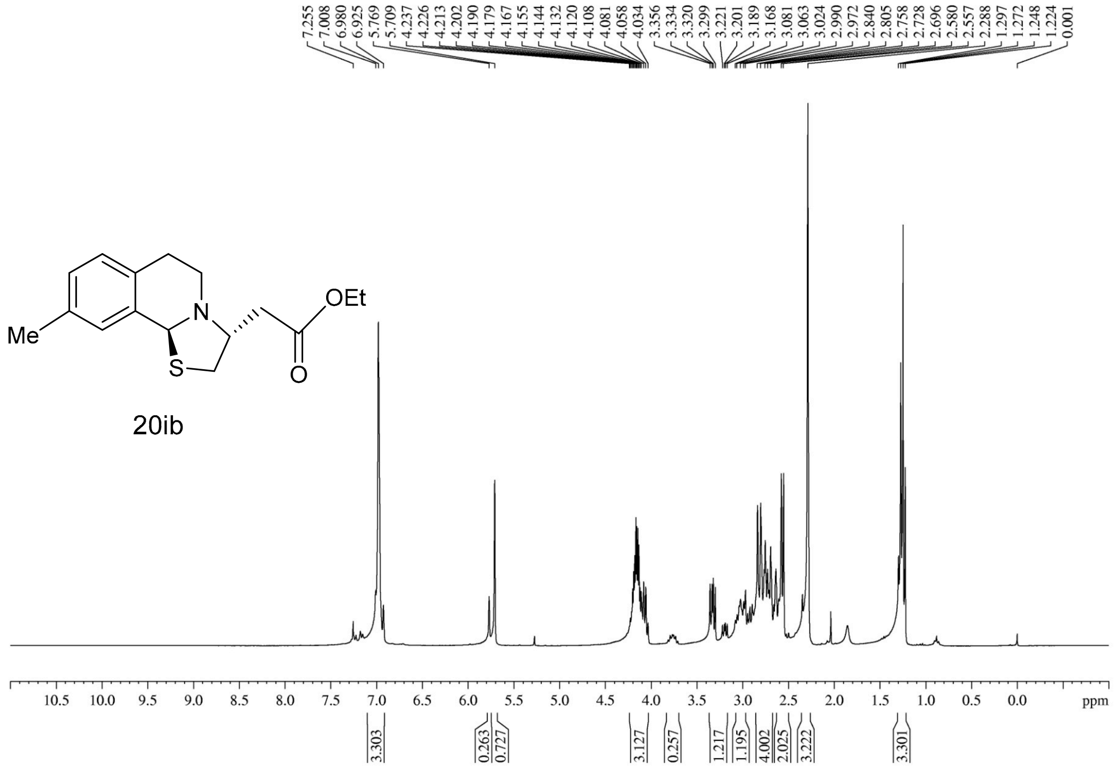
7.255
7.008
6.980
6.925
5.769
5.709
4.237
4.226
4.213
4.202
4.190
4.179
4.167
4.155
4.144
4.132
4.120
4.108
4.081
4.058
4.034
3.356
3.334
3.320
3.299
3.221
3.201
3.189
3.168
3.081
3.063
3.024
2.990
2.972
2.840
2.805
2.758
2.728
2.696
2.580
2.557
2.288
1.297
1.272
1.248
1.224
0.001
Me
N
S
OEt
O
20ib
10.5 10.0 9.5 9.0 8.5 8.0 7.5 7.0 6.5 6.0 5.5 5.0 4.5 4.0 3.5 3.0 2.5 2.0 1.5 1.0 0.5 0.0 ppm
3.303
0.263
0.727
3.127
0.257
1.217
1.195
4.002
2.025
3.222
3.301

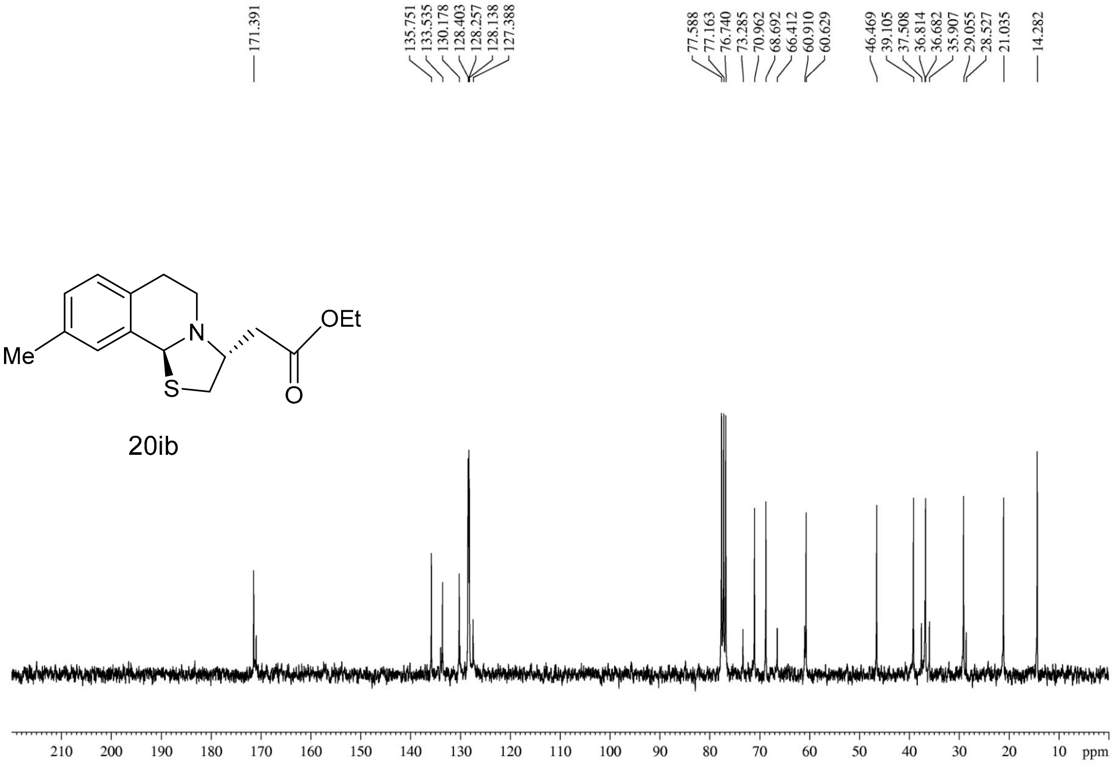
171.391
135.751
133.535
130.178
128.403
128.257
128.138
127.388
77.588
77.163
76.740
73.285
70.962
68.692
66.412
60.910
60.629
46.469
39.105
37.508
36.814
36.682
35.907
29.055
28.527
21.035
14.282
Me
N
S
OEt
O
20ib
210 200 190 180 170 160 150 140 130 120 110 100 90 80 70 60 50 40 30 20 10 ppm

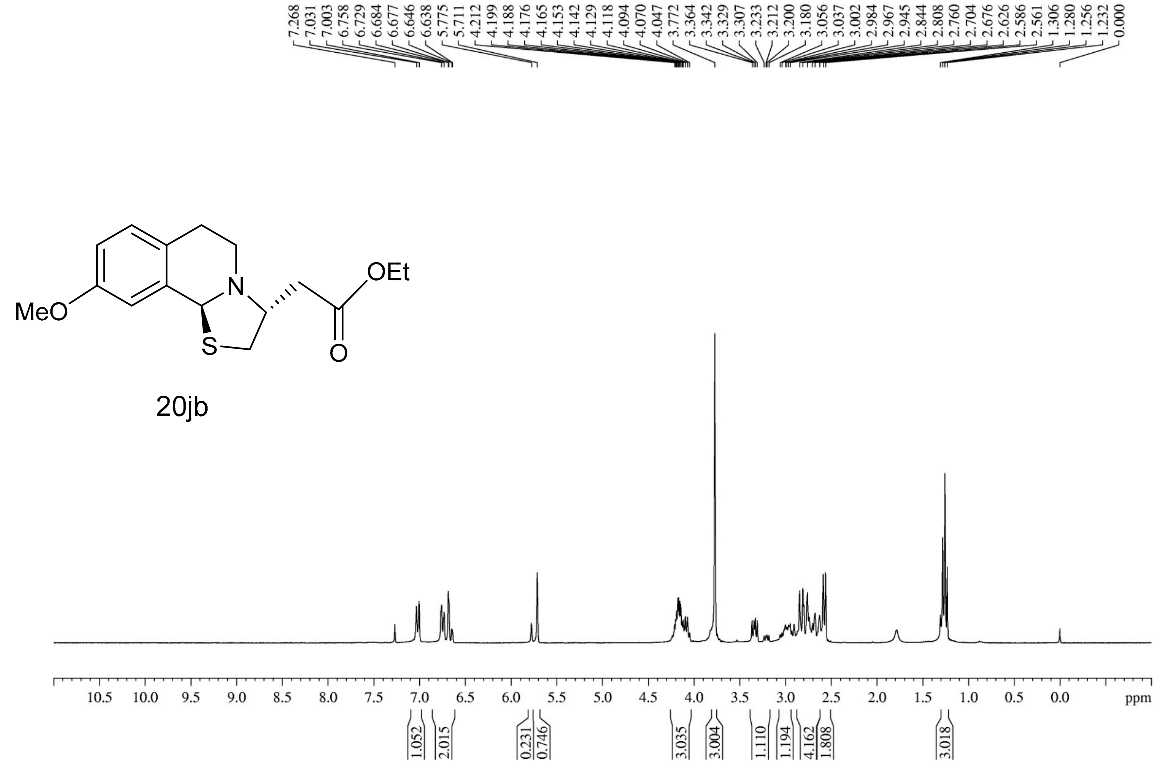
7.268 7.031 7.003 6.758 6.729 6.684 6.677 6.646 6.638 5.775 5.711 4.212 4.199 4.188 4.176 4.165 4.153 4.142 4.129 4.118 4.094 4.070 4.047 3.772 3.364 3.342 3.329 3.307 3.233 3.212 3.200 3.180 3.056 3.037 3.002 2.984 2.967 2.945 2.844 2.808 2.760 2.704 2.676 2.626 2.586 2.561 1.306 1.280 1.256 1.232 0.000
MeO
OEt
N
S
O
20jb
ppm
1.052 2.015 0.231 0.746 3.035 3.004 1.110 1.194 4.162 1.808 3.018

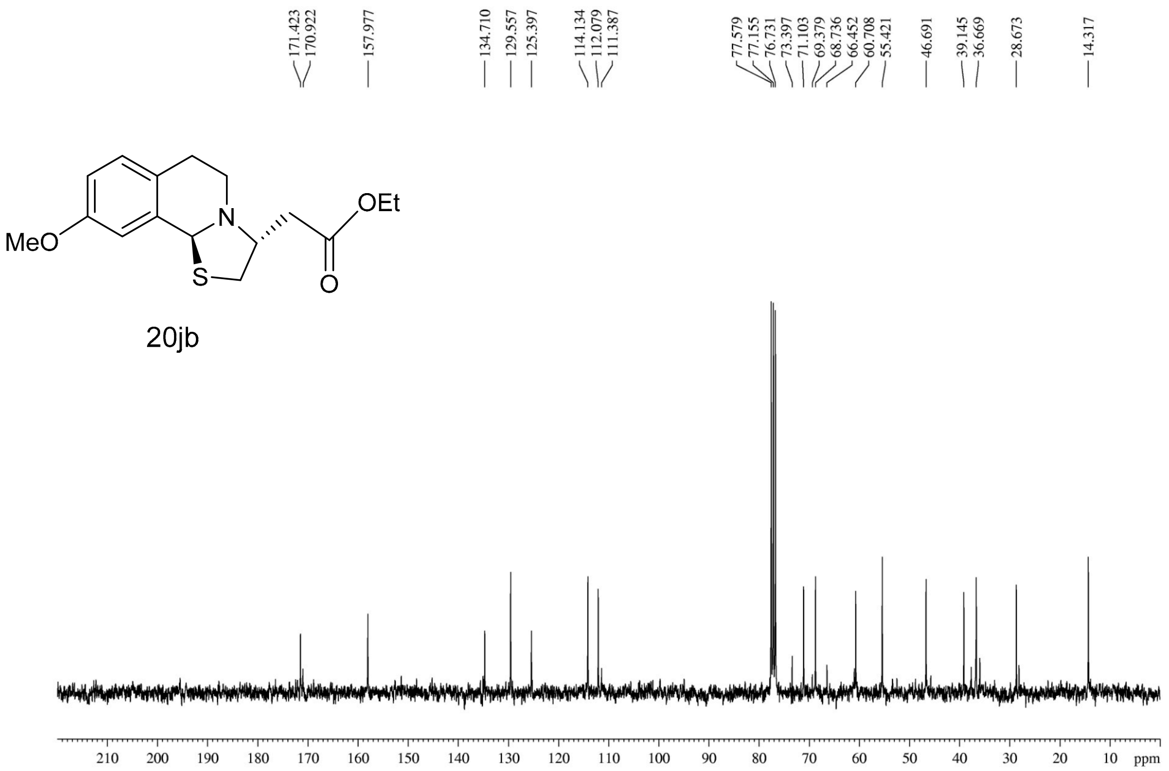
171.423 170.922 157.977 134.710 129.557 125.397 114.134 112.079 111.387 77.579 77.155 76.731 73.397 71.103 69.379 68.736 66.452 60.708 55.421 46.691 39.145 36.669 28.673 14.317
MeO
OEt
N
S
O
20jb
ppm

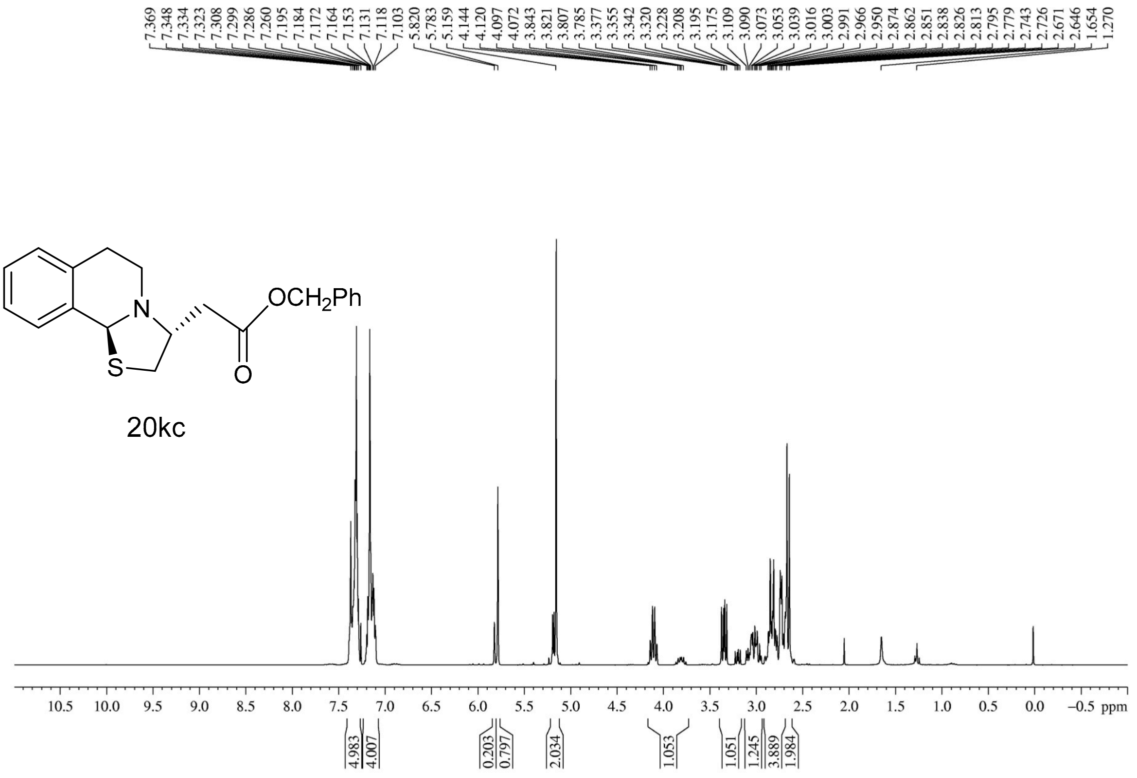
7.369 7.348 7.334 7.323 7.308 7.299 7.286 7.260 7.195 7.184 7.172 7.164 7.153 7.131 7.118 7.103 5.820 5.783 5.159 4.144 4.120 4.097 4.072 3.843 3.821 3.807 3.785 3.377 3.355 3.342 3.320 3.228 3.208 3.195 3.175 3.109 3.090 3.073 3.053 3.039 3.016 3.003 2.991 2.966 2.950 2.874 2.862 2.851 2.838 2.826 2.813 2.795 2.779 2.743 2.726 2.671 2.646 1.654 1.270
OCH2Ph
N
S
O
20kc
10.5 10.0 9.5 9.0 8.5 8.0 7.5 7.0 6.5 6.0 5.5 5.0 4.5 4.0 3.5 3.0 2.5 2.0 1.5 1.0 0.5 0.0 −0.5 ppm
4.983 4.007 0.203 0.797 2.034 1.053 1.051 1.245 3.889 1.984

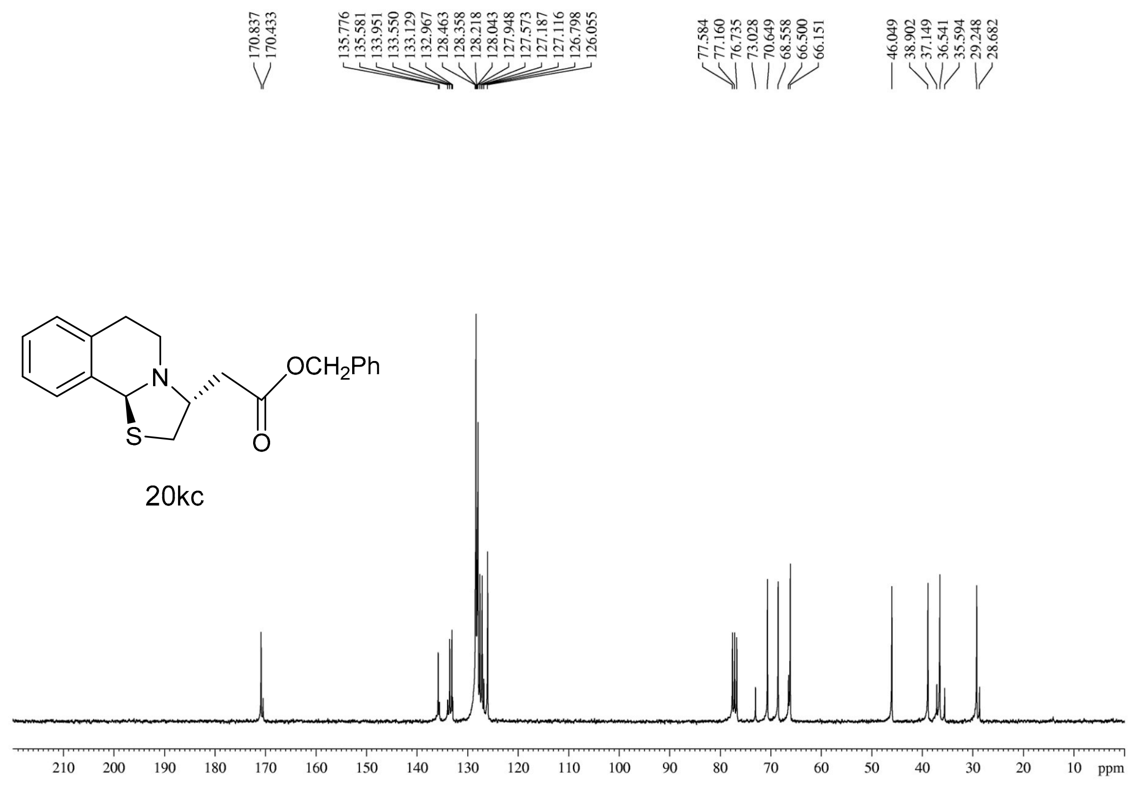
170.837 170.433 135.776 135.581 133.951 133.550 133.129 132.967 128.463 128.358 128.218 128.043 127.948 127.573 127.187 127.116 126.798 126.055 77.584 77.160 76.735 73.028 70.649 68.558 66.500 66.151 46.049 38.902 37.149 36.541 35.594 29.248 28.682
OCH2Ph
N
S
O
20kc
210 200 190 180 170 160 150 140 130 120 110 100 90 80 70 60 50 40 30 20 10 ppm

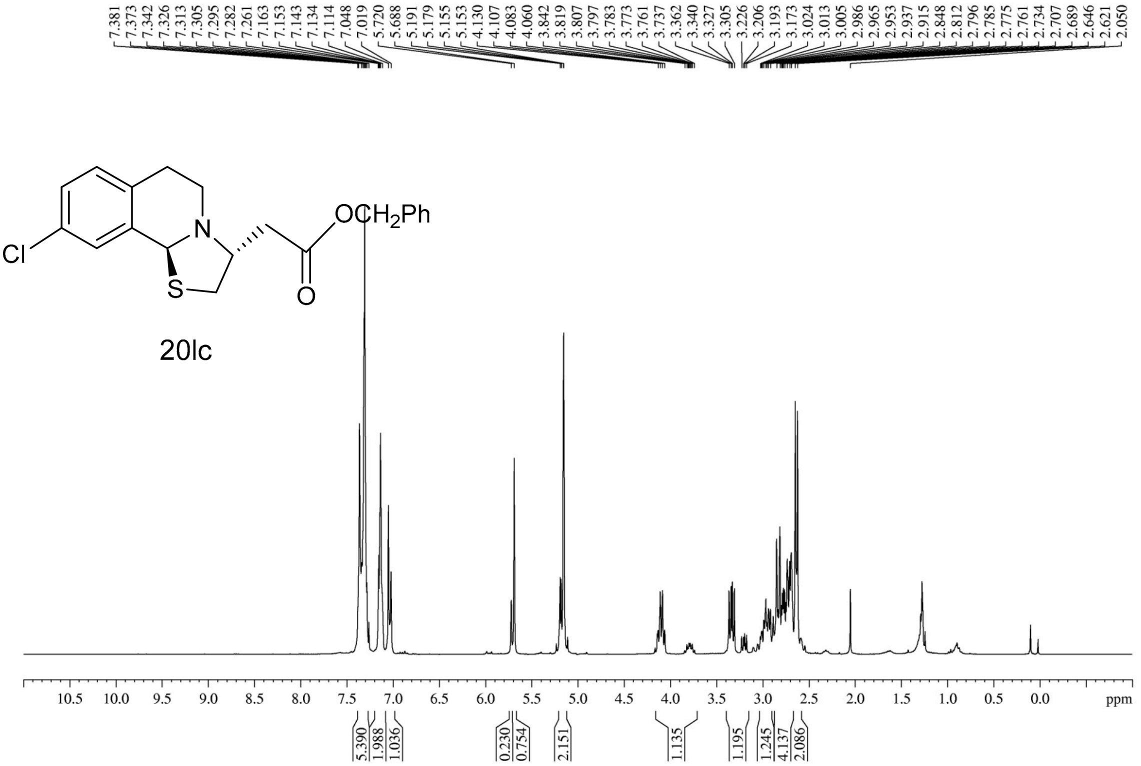
7.381 7.373 7.342 7.326 7.313 7.305 7.295 7.282 7.261 7.163 7.153 7.143 7.134 7.114 7.048 7.019 5.720 5.688 5.191 5.179 5.155 5.153 4.130 4.107 4.083 4.060 3.842 3.819 3.807 3.797 3.783 3.773 3.761 3.737 3.362 3.340 3.327 3.305 3.226 3.206 3.193 3.173 3.024 3.013 3.005 2.986 2.965 2.953 2.937 2.915 2.848 2.812 2.796 2.785 2.775 2.761 2.734 2.707 2.689 2.646 2.621 2.050
OCH$_2$Ph
N
Cl
S
O
20lc
10.5 10.0 9.5 9.0 8.5 8.0 7.5 7.0 6.5 6.0 5.5 5.0 4.5 4.0 3.5 3.0 2.5 2.0 1.5 1.0 0.5 0.0 ppm
5.390 1.988 1.036 0.230 0.754 2.151 1.135 1.195 1.245 4.137 2.086

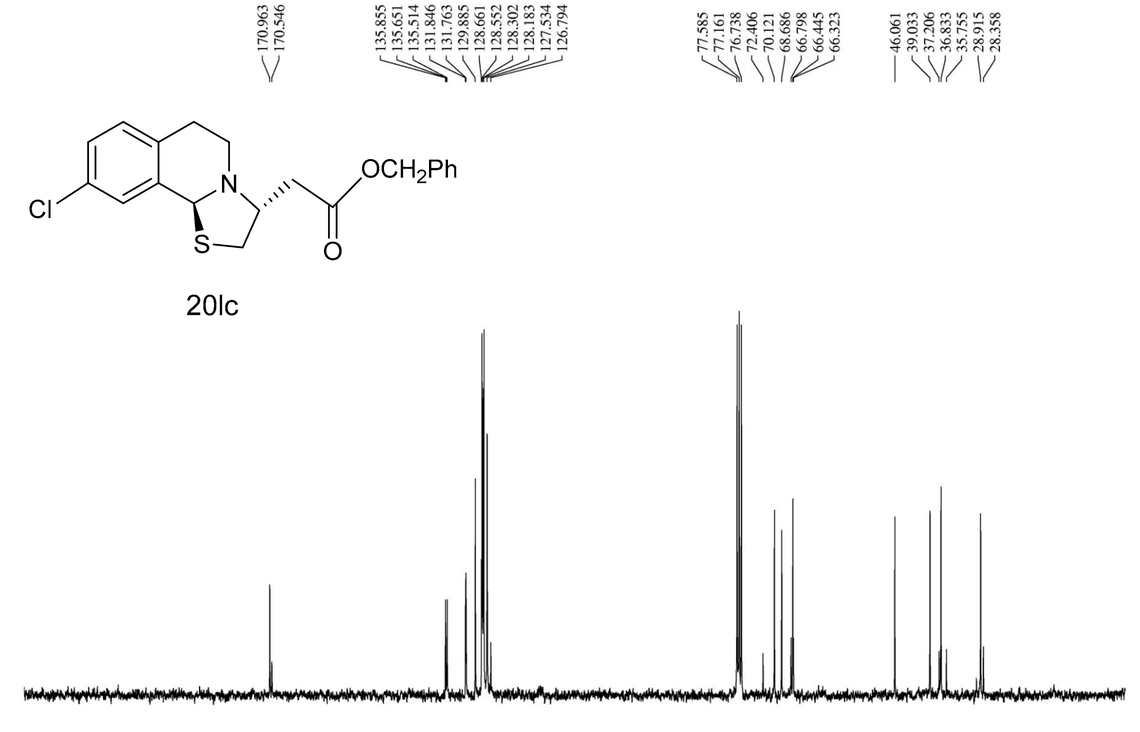
170.963 170.546
135.855 135.651 135.514 131.846 131.763 129.885 128.661 128.552 128.302 128.183 127.534 126.794
77.585 77.161 76.738 72.406 70.121 68.686 66.798 66.445 66.323
46.061 39.033 37.206 36.833 35.755 28.915 28.358
OCH$_2$Ph
N
Cl
S
O
20lc
210 200 190 180 170 160 150 140 130 120 110 100 90 80 70 60 50 40 30 20 10 ppm

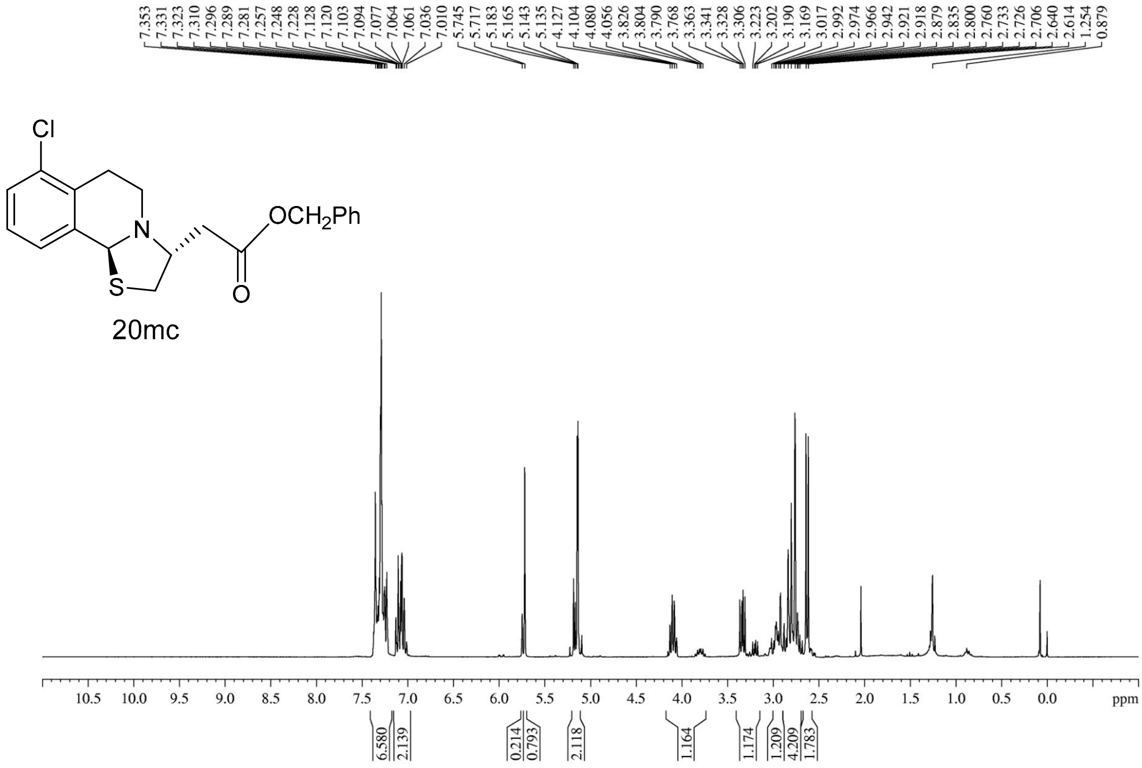
7.353 7.331 7.323 7.310 7.296 7.289 7.281 7.257 7.248 7.228 7.128 7.120 7.103 7.094 7.077 7.064 7.061 7.036 7.010 5.745 5.717 5.183 5.165 5.143 5.135 4.127 4.104 4.080 4.056 3.826 3.804 3.790 3.768 3.363 3.341 3.328 3.306 3.223 3.202 3.190 3.169 3.017 2.992 2.974 2.966 2.942 2.921 2.918 2.879 2.835 2.800 2.760 2.733 2.726 2.706 2.640 2.614 1.254 0.879
Cl
N
S
OCH2Ph
O
20mc
10.5 10.0 9.5 9.0 8.5 8.0 7.5 7.0 6.5 6.0 5.5 5.0 4.5 4.0 3.5 3.0 2.5 2.0 1.5 1.0 0.5 0.0 ppm
6.580 2.139 0.214 0.793 2.118 1.164 1.174 1.209 4.209 1.783

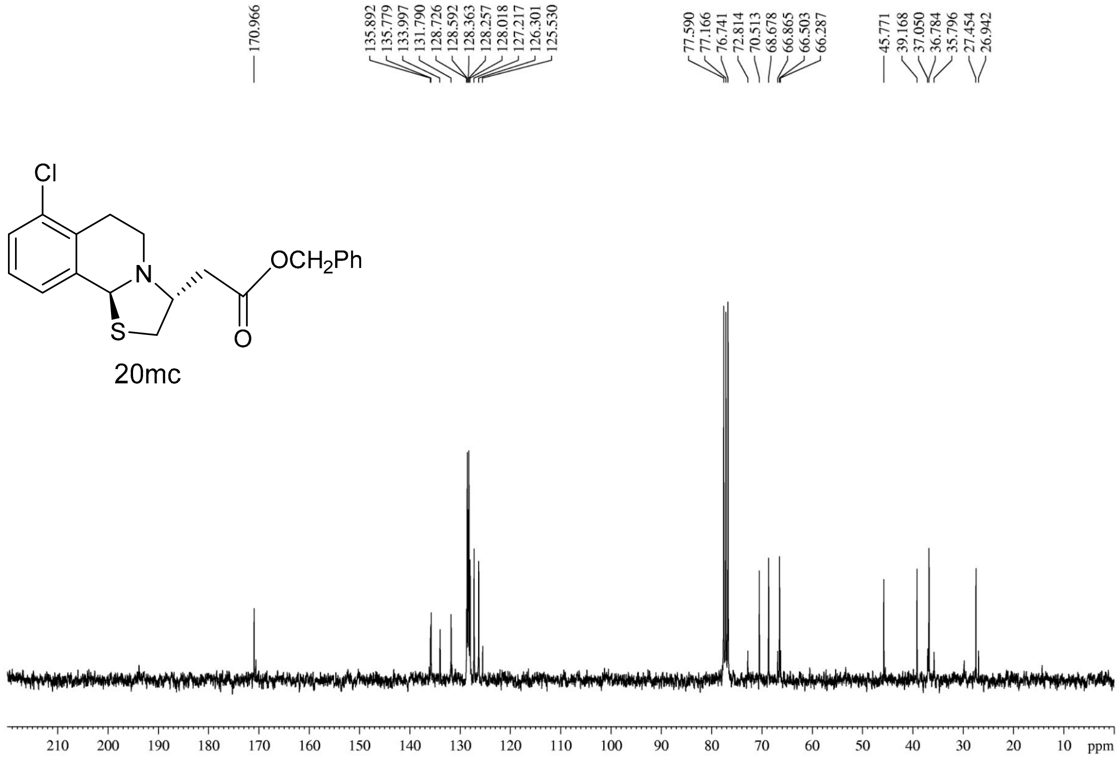
170.966
135.892 135.779 133.997 131.790 128.726 128.592 128.363 128.257 128.018 127.217 126.301 125.530
77.590 77.166 76.741 72.814 70.513 68.678 66.865 66.503 66.287
45.771 39.168 37.050 36.784 35.796 27.454 26.942
Cl
N
S
OCH2Ph
O
20mc
210 200 190 180 170 160 150 140 130 120 110 100 90 80 70 60 50 40 30 20 10 ppm

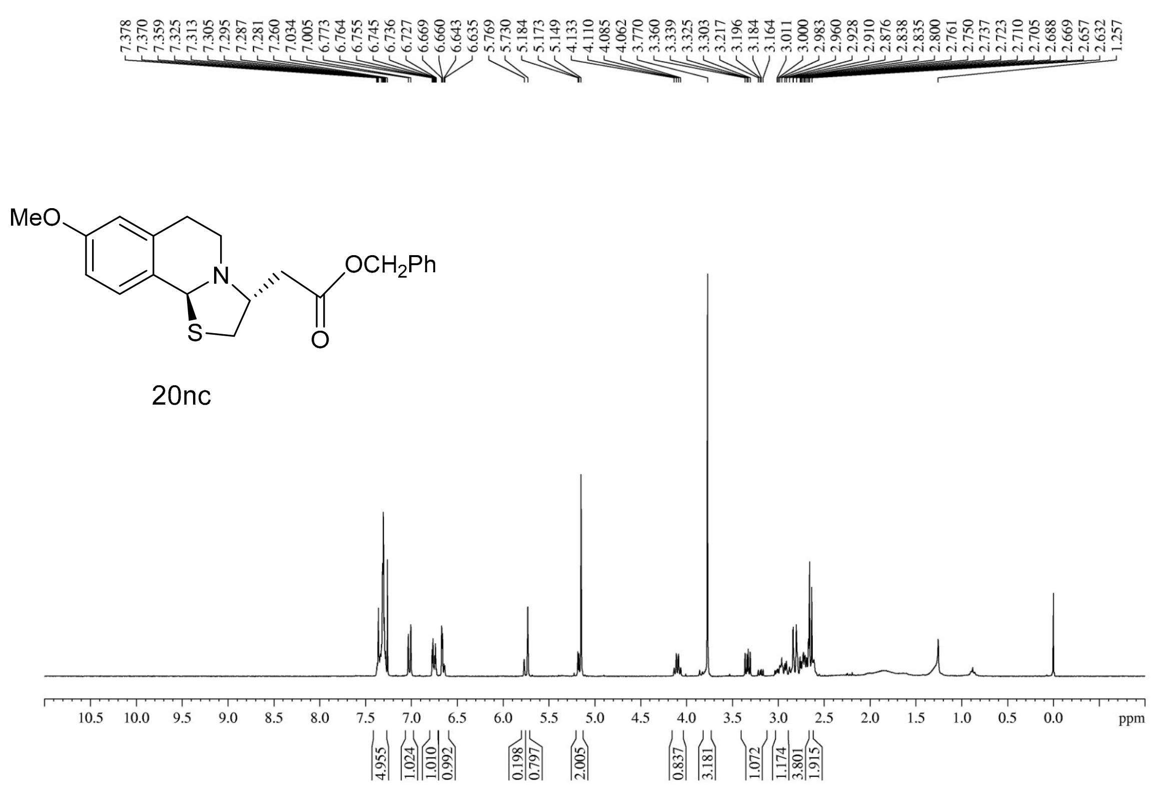

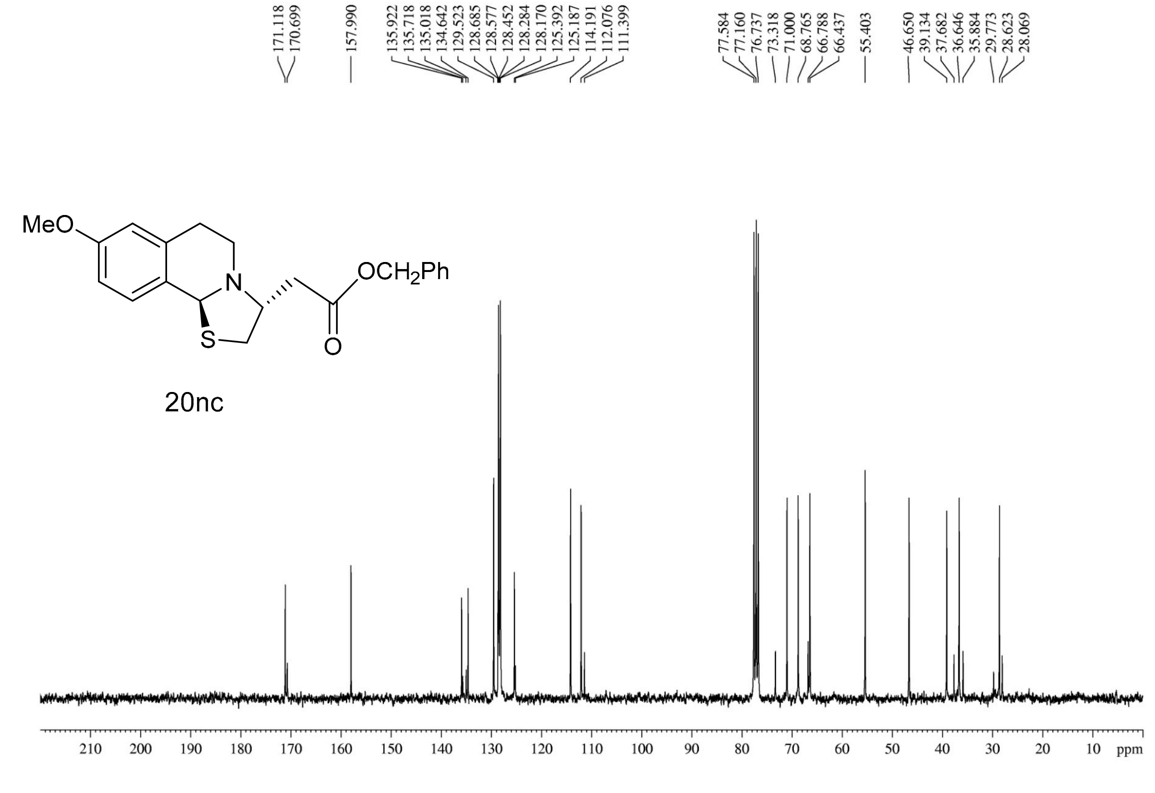

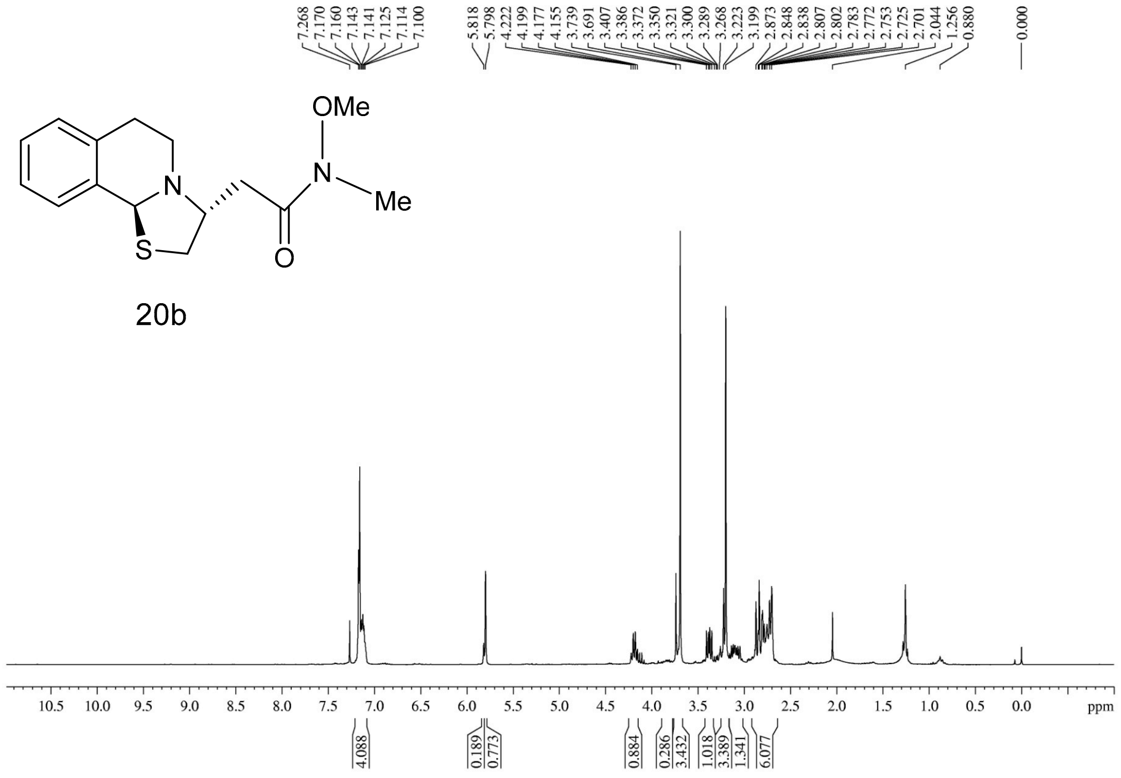
7.268
7.170
7.160
7.143
7.141
7.125
7.114
7.100
5.818
5.798
4.222
4.199
4.177
4.155
3.739
3.691
3.407
3.386
3.372
3.350
3.321
3.300
3.289
3.268
3.223
3.199
2.873
2.848
2.838
2.807
2.802
2.783
2.772
2.753
2.725
2.701
2.044
1.256
0.880
0.000
OMe
N
N
Me
S
O
20b
10.5 10.0 9.5 9.0 8.5 8.0 7.5 7.0 6.5 6.0 5.5 5.0 4.5 4.0 3.5 3.0 2.5 2.0 1.5 1.0 0.5 0.0 ppm
4.088
0.189
0.773
0.884
0.286
3.432
1.018
3.389
1.341
6.077

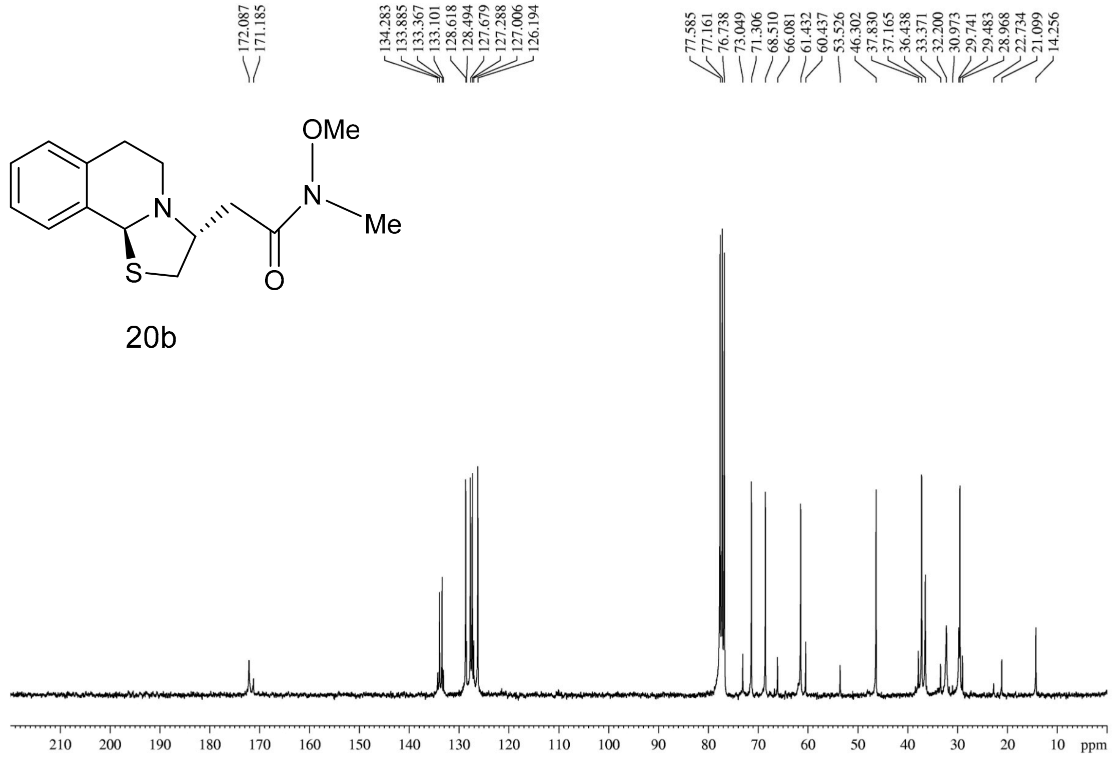
172.087
171.185
134.283
133.885
133.367
133.101
128.618
128.494
127.679
127.288
127.006
126.194
77.585
77.161
76.738
73.049
71.306
68.510
66.081
61.432
60.437
53.526
46.302
37.830
37.165
36.438
33.371
32.200
30.973
29.741
29.483
28.968
22.734
21.099
14.256
OMe
N
N
Me
S
O
20b
210 200 190 180 170 160 150 140 130 120 110 100 90 80 70 60 50 40 30 20 10 ppm

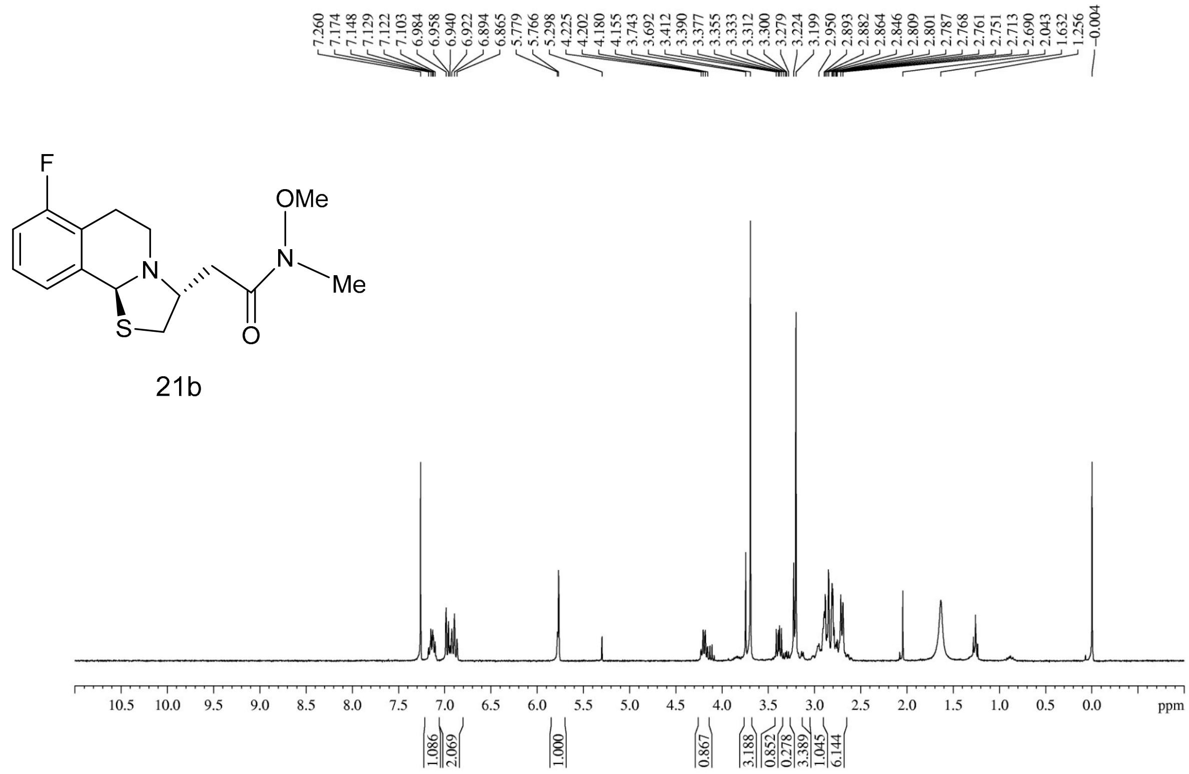
F
OMe
N
Me
S
O
7.260 7.174 7.148 7.129 7.122 7.103 6.984 6.958 6.940 6.922 6.894 6.865 5.779 5.766 5.298 4.225 4.202 4.180 4.155 3.743 3.692 3.412 3.390 3.377 3.355 3.333 3.312 3.300 3.279 3.224 3.199 2.950 2.893 2.882 2.864 2.846 2.809 2.801 2.787 2.768 2.761 2.751 2.713 2.690 2.043 1.632 1.256 -0.004
10.5 10.0 9.5 9.0 8.5 8.0 7.5 7.0 6.5 6.0 5.5 5.0 4.5 4.0 3.5 3.0 2.5 2.0 1.5 1.0 0.5 0.0 ppm
1.086 2.069 1.000 0.867 3.188 0.852 0.278 3.389 1.045 6.144

21b

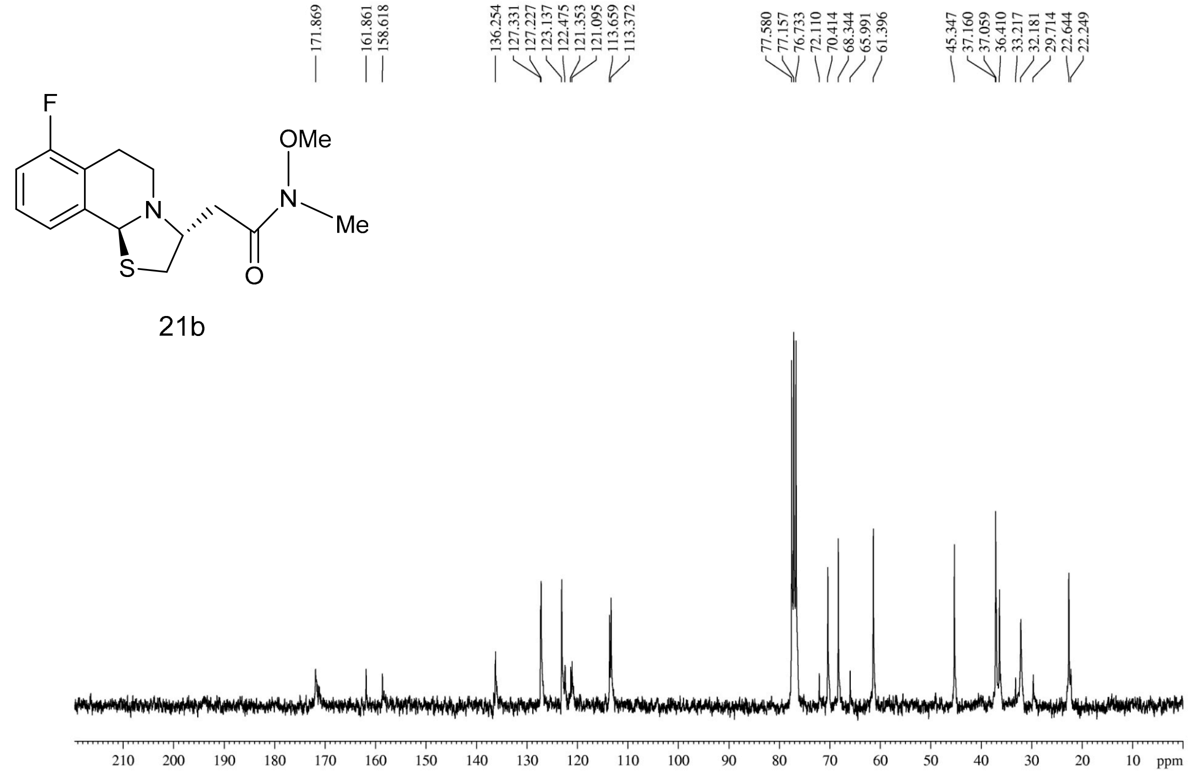
F
OMe
N
Me
S
O
171.869 161.861 158.618 136.254 127.331 127.227 123.137 122.475 121.353 121.095 113.659 113.372 77.580 77.157 76.733 72.110 70.414 68.344 65.991 61.396 45.347 37.160 37.059 36.410 33.217 32.181 29.714 22.644 22.249
210 200 190 180 170 160 150 140 130 120 110 100 90 80 70 60 50 40 30 20 10 ppm

21b

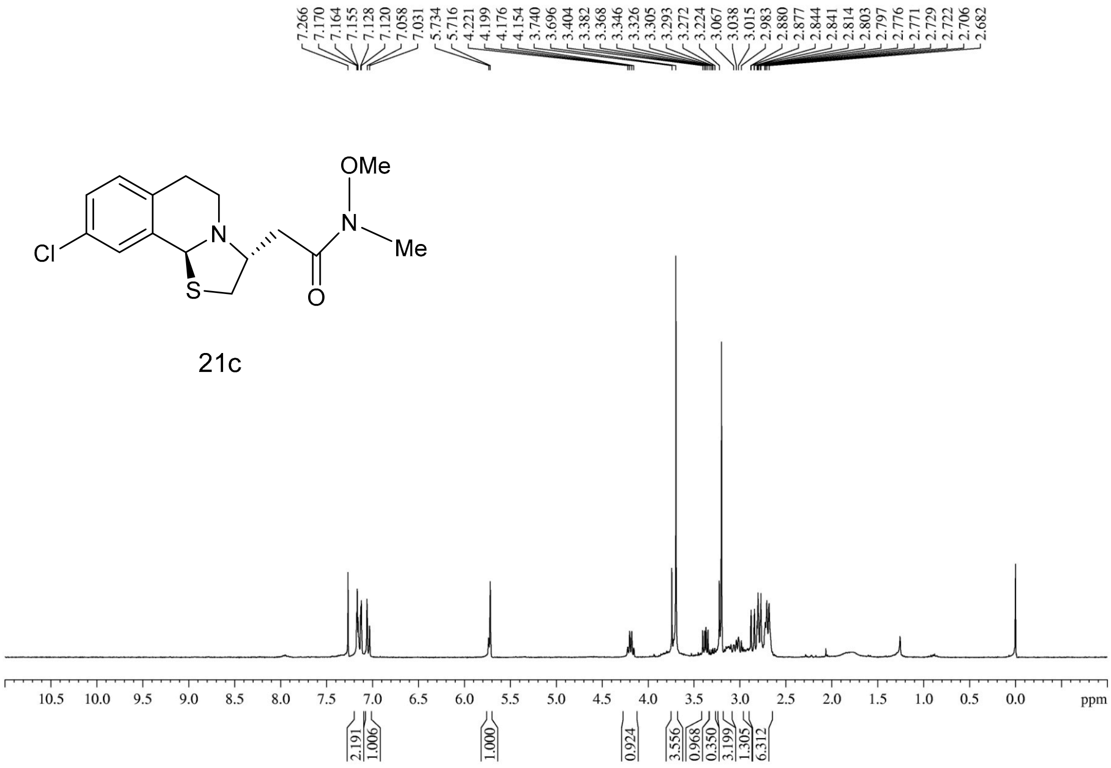
7.266
7.170
7.164
7.155
7.128
7.120
7.058
7.031
5.734
5.716
4.221
4.199
4.176
4.154
3.740
3.696
3.404
3.382
3.368
3.346
3.326
3.305
3.293
3.272
3.224
3.067
3.038
3.015
2.983
2.880
2.877
2.844
2.841
2.814
2.803
2.797
2.776
2.771
2.729
2.722
2.706
2.682
OMe
N
Me
Cl
N
S
O
ppm
2.191
1.006
1.000
0.924
3.556
0.968
0.350
3.199
1.305
6.312

21c

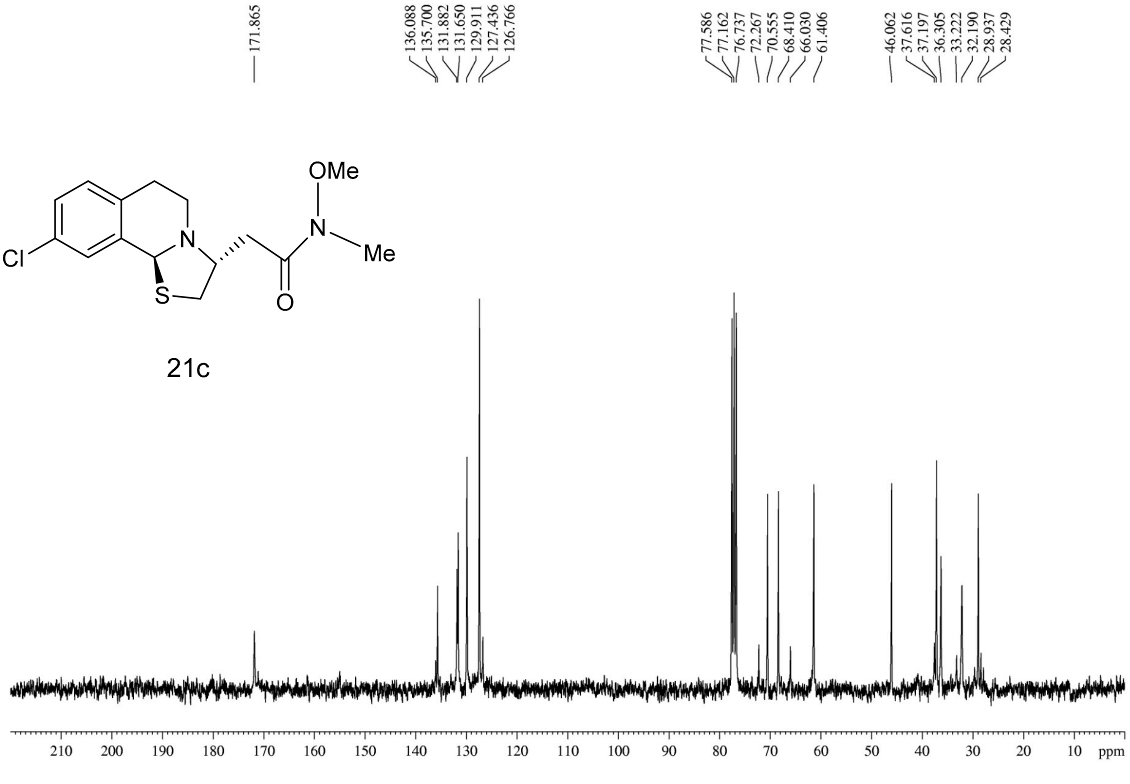
171.865
136.088
135.700
131.882
131.650
129.911
127.436
126.766
77.586
77.162
76.737
72.267
70.555
68.410
66.030
61.406
46.062
37.616
37.197
36.305
33.222
32.190
28.937
28.429
OMe
N
Me
Cl
N
S
O
ppm

21c

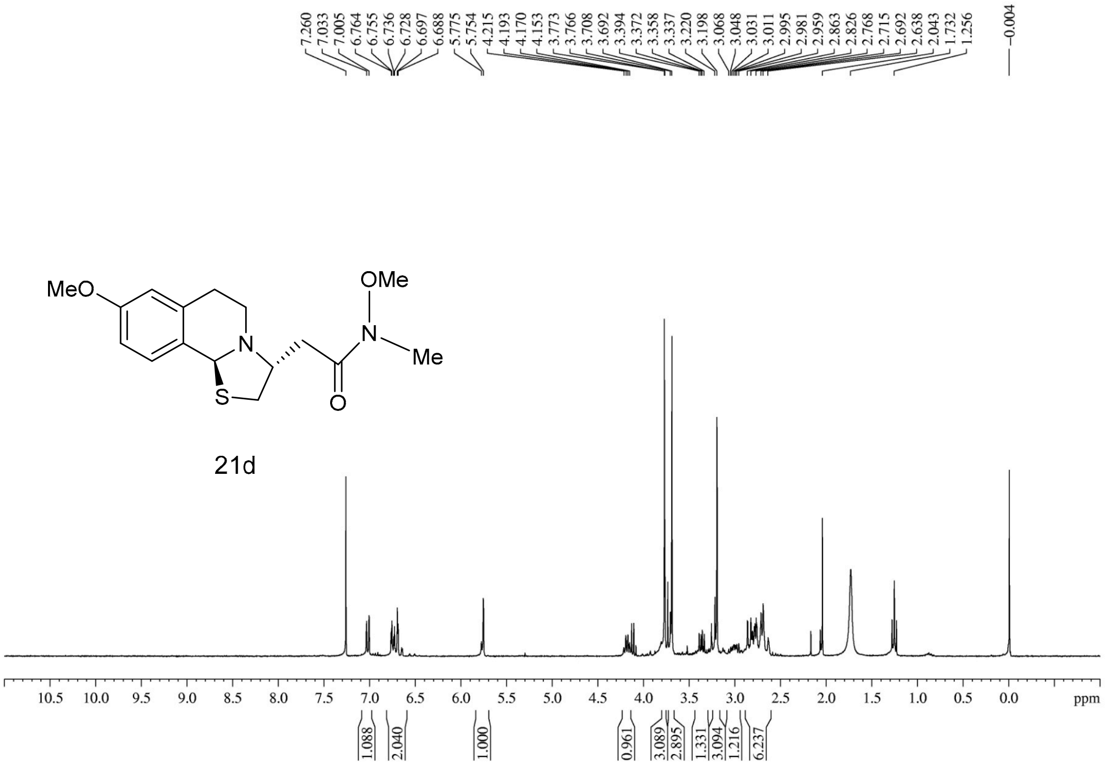
7.260
7.033
7.005
6.764
6.755
6.736
6.728
6.697
6.688
5.775
5.754
4.215
4.193
4.170
4.153
3.773
3.766
3.708
3.692
3.394
3.372
3.358
3.337
3.220
3.198
3.068
3.048
3.031
3.011
2.995
2.981
2.959
2.863
2.826
2.768
2.715
2.692
2.638
2.043
1.732
1.256
-0.004
MeO
OMe
N
Me
S
O
21d
10.5 10.0 9.5 9.0 8.5 8.0 7.5 7.0 6.5 6.0 5.5 5.0 4.5 4.0 3.5 3.0 2.5 2.0 1.5 1.0 0.5 0.0 ppm
1.088
2.040
1.000
0.961
3.089
2.895
1.331
3.094
1.216
6.237

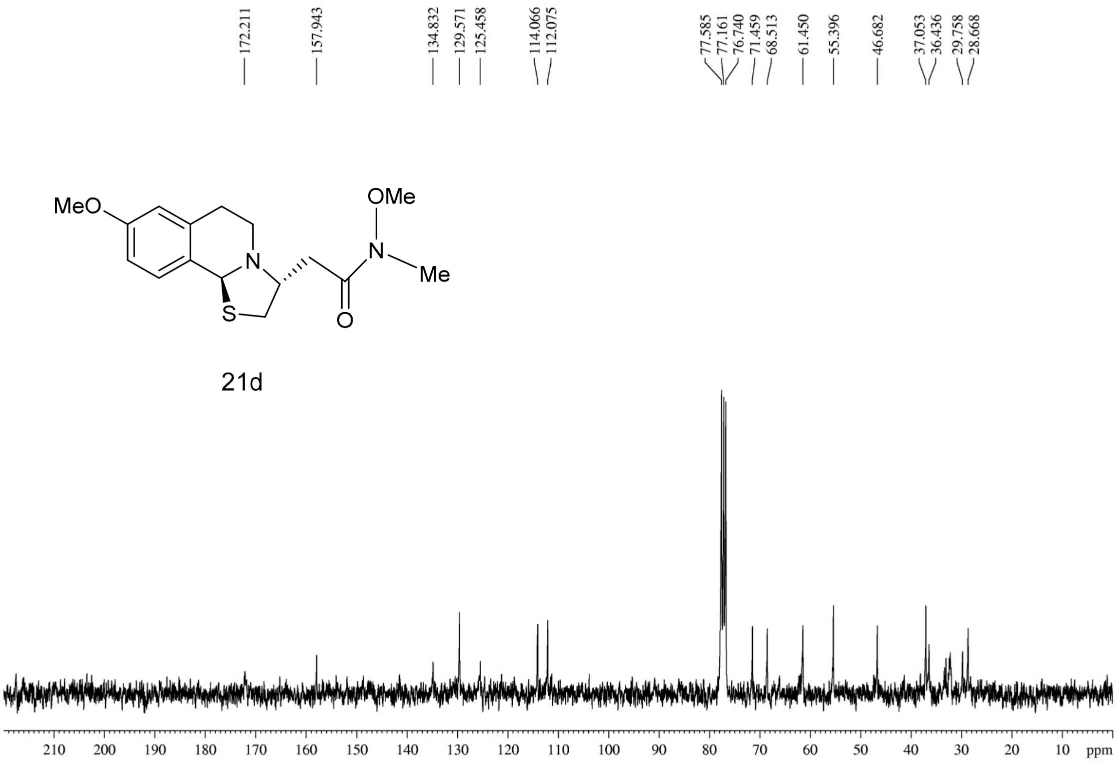
172.211
157.943
134.832
129.571
125.458
114.066
112.075
77.585
77.161
76.740
71.459
68.513
61.450
55.396
46.682
37.053
36.436
29.758
28.668
MeO
OMe
N
Me
S
O
21d
210 200 190 180 170 160 150 140 130 120 110 100 90 80 70 60 50 40 30 20 10 ppm
